# Comparative phenotypic and transcriptomic analyses unravel conserved and distinct mechanisms underlying shade avoidance syndrome in Brassicaceae vegetables

**DOI:** 10.1186/s12864-021-08076-1

**Published:** 2021-10-25

**Authors:** Nguyen Hoai Nguyen, Benny Jian Rong Sng, Hock Chuan Yeo, In-Cheol Jang

**Affiliations:** 1 Temasek Life Sciences Laboratory, 1 Research Link, National University of Singapore, Singapore, 117604 Singapore; 2 Department of Biological Sciences, National University of Singapore, Singapore, 117543 Singapore

**Keywords:** Auxin, *Brassica oleracea*, *Brassica rapa*, RNA-seq, de novo transcriptome assembly, Phytohormones

## Abstract

**Background:**

Plants grown under shade are exposed to low red/far-red ratio, thereby triggering an array of altered phenotypes called shade avoidance syndrome (SAS). Shade negatively influences plant growth, leading to a reduction in agricultural productivity. Understanding of SAS is crucial for sustainable agricultural practices, especially for high-density indoor farming. Brassicaceae vegetables are widely consumed around the world and are commonly cultivated in indoor farms. However, our understanding of SAS in Brassicaceae vegetables and their genome-wide transcriptional regulatory networks are still largely unexplored.

**Results:**

Shade induced common signs of SAS, including hypocotyl elongation and reduced carotenoids/anthocyanins biosynthesis, in two different Brassicaceae species: *Brassica rapa* (Choy Sum and Pak Choy) and *Brassica oleracea* (Kai Lan). Phenotype-assisted transcriptome analysis identified a set of genes induced by shade in these species, many of which were related to auxin biosynthesis and signaling [e.g. *YUCCA8* (*YUC8*), *YUC9*, and *INDOLE-3-ACETIC ACID INDUCIBLE* (*IAAs*)] and other phytohormones signaling pathways including brassinosteroids and ethylene. The genes functioning in plant defense (e.g. *MYB29* and *JASMONATE-ZIM-DOMAIN PROTEIN 9*) as well as in biosynthesis of anthocyanins and glucosinolates were repressed upon shade. Besides, each species also exhibited distinct SAS phenotypes. Shade strongly reduced primary roots and elongated petioles of *B. oleracea,* Kai Lan. However, these SAS phenotypes were not clearly recognized in *B. rapa*, Choy Sum and Pak Choy. Some auxin signaling genes (e.g. *AUXIN RESPONSE FACTOR 19*, *IAA10*, and *IAA20*) were specifically induced in *B. oleracea*, while homologs in *B. rapa* were not up-regulated under shade. Contrastingly, shade-exposed *B. rapa* vegetables triggered the ethylene signaling pathway earlier than *B. oleracea*, Kai Lan. Interestingly, shade induced the transcript levels of *LONG HYPOCOTYL IN FAR-RED 1* (*HFR1*) homolog in only Pak Choy as *B. rapa*. As HFR1 is a key negative regulator of SAS in Arabidopsis, our finding suggests that Pak Choy HFR1 homolog may also function in conferring higher shade tolerance in this variety.

**Conclusions:**

Our study shows that two Brassicaceae species not only share a conserved SAS mechanism but also exhibit distinct responses to shade, which will provide comprehensive information to develop new shade-tolerant cultivars that are suitable for high-density indoor farms.

**Supplementary Information:**

The online version contains supplementary material available at 10.1186/s12864-021-08076-1.

## Background

In nature, shade occurs under plant canopy or when many plants germinate and grow in confined spaces such as during high-density farming. As compared to open canopies, plants growing under shade receive more far-red (FR) wavelength reflected from neighboring plants or high trees, since photosynthetic pigments in leaves of neighboring plants selectively absorb red (R) and blue (B) [1]. This reduces the total light intensity and R/FR ratio, which change plant development and exhibit a collection of phenotypes called shade avoidance syndrome or response (SAS or SAR) [2]. Typically, SAS includes elongated growth of stem and leaf petiole, increased apical dominance, and early flowering [2–4].

SAS has been widely studied in the model plant *Arabidopsis thaliana* [5]. Among Arabidopsis photoreceptors, phytochrome B (phyB) has been documented as a major regulator of SAS because its mutant displays constitutive SAS phenotype including elongated hypocotyl and petiole, defect in root growth, and early flowering [6, 7]. The nuclei-located active phyB promotes photomorphogenesis by inhibiting the activities of PHYTOCHROME-INTERACTING FACTORS (PIFs) through a direct interaction [8, 9]. PIFs are transcription factors that bind to the promoters of shade-induced genes and activate their expression, thereby causing SAS [10]. The phyB-bound PIFs can be targeted for degradation by 26S proteasomes [9, 11]. Under a FR-enriched environment like shade, active phyB reverts to its inactive form, which causes stabilizing PIFs to trigger a signaling cascade that leads to SAS [9, 12]. Some PIF proteins (PIF4, PIF5, and PIF7) have been found to directly activate the transcription of several auxin biosynthetic genes such as *TRYPTOPHAN AMINOTRANSFERASE OF ARABIDOPSIS1* (*TAA1*) and *YUCCAs* (*YUCs*) [10, 13, 14]. It is well-known that phytohormone auxin mediates low R/FR-induced cell elongation in Arabidopsis [13]. In response to shade, auxin biosynthesis, auxin polar transport, and plant sensitivity to auxin are quickly activated to facilitate the hypocotyl and petiole elongation [4]. Under deep shade condition, activated phytochrome A (phyA) prevents degradation of auxin signaling repressors, auxin/indole-3-acetic acid proteins (AUX/IAA) through the direct interaction with auxin receptor, TRANSPORT INHIBITOR RESISTANT 1 (TIR1), which leads to a reduction of SAS [15].

In addition to Arabidopsis, SAS has been also studied in different plant species such as tomato, maize, and even conifers, pine and spruce [16–18]. The phenotypic and transcriptomic analyses of two gymnosperm species, pine and spruce showed that there are some conserved as well as different aspects of SAS between angiosperms and gymnosperms [18]. Additionally, this study also suggested that the shade responses in both conifer species, Scots pine and Norway spruce, could be involved in carbon re-allocation [18]. Upon shade, the shade-tolerant Norway spruce decreased biomass production to catch the light better whereas the shade-sensitive Scots pine increased the tissue elongation to reach the light source [18]. Being different from sunlight, light-emitting diodes (LEDs), an artificial light source commonly used for indoor farming, do not generally contain FR. A previous study on tomato found that supplementation of FR increased the number of fruits as well as total fruit fresh weight in comparison to a control condition [19]. In Brassicaceae family, Arabidopsis is closely related to other Brassicaceae vegetables such as *Brassica oleracea* and *Brassica rapa* [20]. Fresh or preserved Brassicaceae vegetables are consumed in the human diet, sometimes as a source of cooking oil and condiments [21]. Natural compounds such as glucosinolates, polyphenols, and triterpenes that are beneficial to human health have been identified in various Brassicaceae vegetables [21, 22]. Shade causes a reduction in growth performance and productivity of crops. For this reason, farmers control spacing for interplanting of crops to maximize the productivity. As high-density planting inevitably results in shaded environments that cause SAS, studying on the mechanism of shade or low-light responses in plants will provide fundamental ideas to develop shade-tolerant crop cultivars and novel technologies to improve the productivity in limited agricultural space.

In this study, two different Brassicaceae species, *B. rapa* (Choy Sum and Pak Choy) and *B. oleracea* (Kai Lan) were used to characterize their growth and responses under shade condition. Morphological analysis indicated that Brassicaceae vegetables exhibited typical SAS phenotypes such as elongated hypocotyl and reduction of leaf blade area that can be also found in Arabidopsis. Interestingly, different species of Brassicaceae vegetables showed some distinct phenotypic responses to shade. To understand the molecular mechanism of SAS behind different phenotypic responses to shade in these vegetables, RNA sequencing (RNA-seq) and de novo transcriptome assembly were performed for these Brassicaceae species (all three vegetables) grown under normal light and simulated shade conditions. Comprehensive transcriptomic analysis identified auxin- and defense-related genes to be up- and down-regulated, respectively, in three vegetables grown under shade. Moreover, we found that each vegetable contained different auxin basal levels and the accumulation of this phytohormone was dramatically elevated in response to shade treatment. Taken together, our results provide an extensive understanding of the conserved and distinct SAS mechanisms in different Brassicaceae species.

## Results

### SAS of Brassicaceae vegetables

**Fig. 1:**
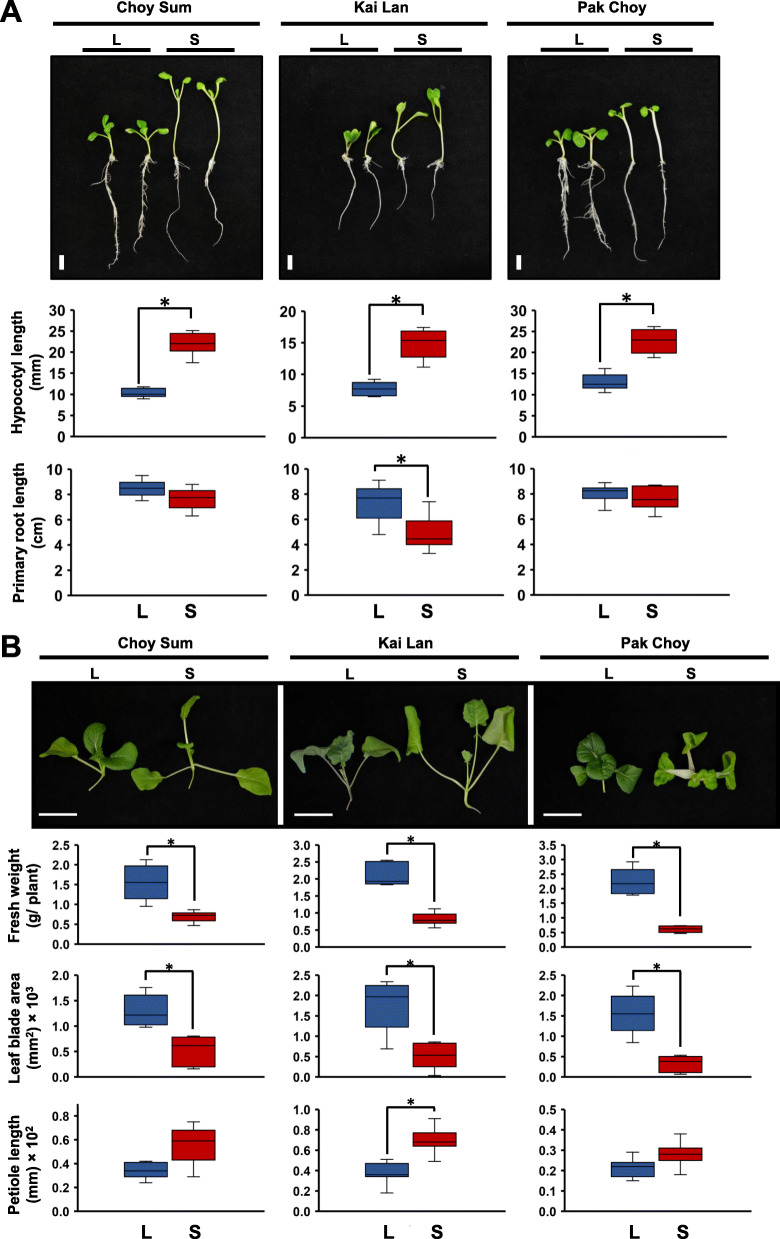
Phenotypic analysis of Brassicaceae vegetables under shade. **a** Four-day-old seedlings grown on MS medium under normal white light (L) were either kept under L or simulated shade (S) for one week. Hypocotyl and primary root were measured after one week of treatments. Scale = 1 cm. **b** Two-week-old vegetables grown on soil under L were either kept under L or S for one week. Scale = 5 cm. Bars represent average ± SD. *n* = 10. Columns marked with an asterisk differ significantly (*p-value* < 0.05)

**Fig. 2:**
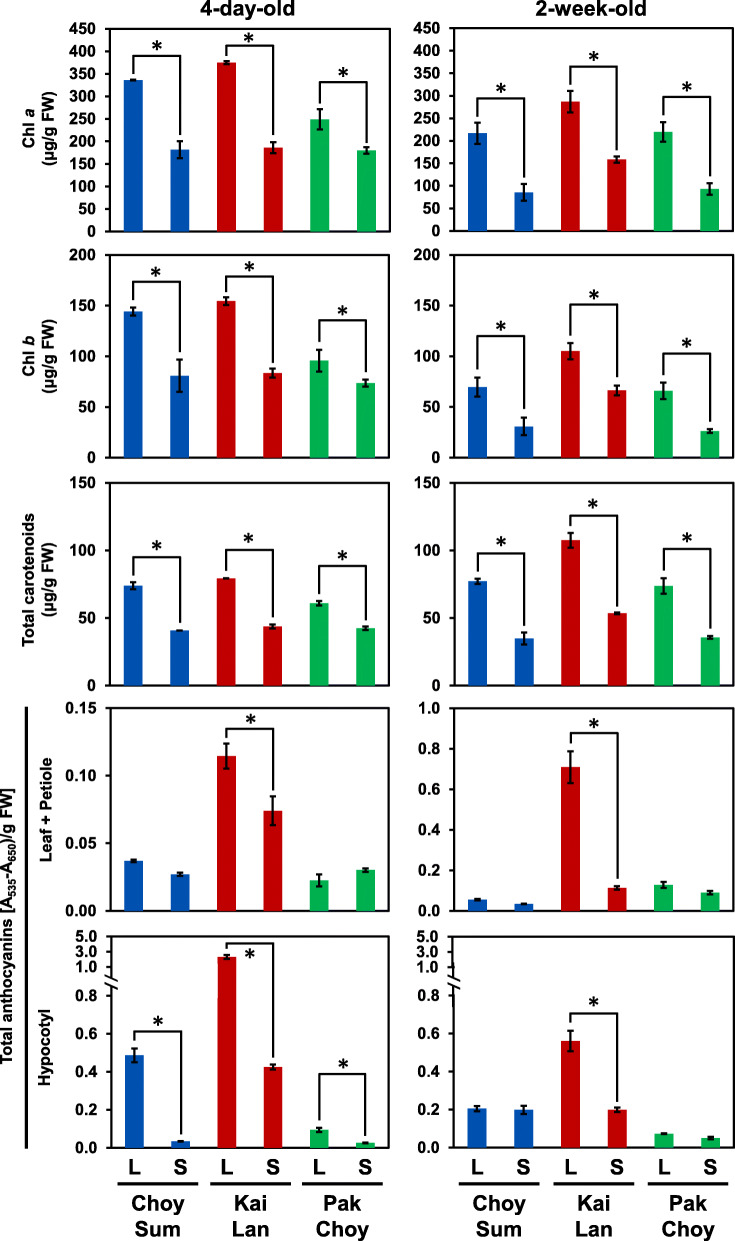
The change of Chl *a*/*b*, total carotenoids, and total anthocyanins levels in Brassicaceae vegetables under shade. Leaves, petioles, or hypocotyls of three Brassicaceae vegetables, Choy Sum, Kai Lan, and Pak Choy, mentioned in Fig. 1 were used for quantification of Chl *a/b*, total carotenoids, and total anthocyanins. Bars represent average ± SD. *n* = 3. Columns marked with an asterisk differ significantly (*p-value* < 0.05)

Two different Brassicaceae species, *B. rapa* (Choy Sum and Pak Choy) and *B. oleracea* (Kai Lan) were tested for their growth performance in response to shade. Figure 1a shows that the hypocotyls of 4-day-old Brassicaceae vegetables were significantly elongated under simulated shade condition (S) [low R/FR ratio and low photosynthetic active radiation (PAR)]. Accordingly, the hypocotyl cell sizes of shade-exposed seedlings were also longer than those of normal growth seedlings (Fig. S1 in Additional file 1). Besides, shade also reduced the lateral root growth in these vegetables (Fig. S2 in Additional file 1). However, only *B. oleracea* Kai Lan exhibited a significant reduction in primary root elongation while the other *B. rapa* vegetables, Choy Sum and Pak Choy did not change their primary root length in response to shade (Fig. 1a). These shade-induced phenotypes were more obvious in 2-week-old vegetables having true leaves (Fig. 1b). Shade highly reduced total fresh weight and leaf size of the Brassicaceae vegetables (Fig. 1b). Under shade condition, *B. oleracea* Kai Lan dramatically elongated while *B. rapa* Choy Sum also somewhat extended their leaf petiole (Fig. 1b). Nevertheless, shade-exposed *B. rapa* Pak Choy only lengthened its leaf petiole slightly (Fig. 1b). In addition to these typical SAS phenotypes of Brassicaceae species, shade also reduced the accumulation of Chlorophyll *a/b* (Chl *a*/*b*), total carotenoids, and total anthocyanins in both 4-day-old and 2-week-old plants (Fig. 2). Overall, these data indicate that shade undoubtedly results in the development of typical SAS such as elongated hypocotyl and reduced leaf expansion in Brassicaceae species. However, each species shows slightly different responses upon shade treatment.

### RNA-seq and de novo transcriptome assembly

To investigate the molecular mechanism of SAS in Brassicaceae species, we performed RNA-seq of 2-week-old plants after exposure to different durations of shade (1 and 6 h). More than 96–183 million reads were obtained from each sample (Table S1 in Additional file 1). The average GC% for Choy Sum, Kai Lan, and Pak Choy was approximately 48.19, 47.95, and 48.12%, respectively (Table S1 in Additional file 1). RNA-seq data from each sample was highly qualified by the Phred quality scores showing over 98% (Q20) and 95% (Q30) (Table S1 in Additional file 1).

After de novo assembly of total reads using Trinity, we obtained 156,234, 189,879, and 175,656 of assembled transcript contigs with the N50 values of 1227, 1213, and 1196 (bp) from Choy Sum, Kai Lan, and Pak Choy, respectively (Table S2 in Additional file 1). These assembled transcript contigs were then filtered and clustered into the non-redundant transcript contigs (unigenes). As a result, 86,733, 99,896, and 95,984 unigenes were procured from Choy Sum, Kai Lan, and Pak Choy, respectively (Table S3 in Additional file 1). These unigenes were used for further analyses including functional annotation and differentially expressed genes (DEGs).

### Functional annotation of unigenes

For functional annotation of unigenes, assembled transcript contigs were processed using Basic Local Alignment Search Tool (BLAST) against sequences in several public databases including Gene ontology (GO), UniProt, National Center for Biotechnology Information (NCBI) non-redundant Protein (NR), Protein families (Pfam), evolutionary genealogy of genes, Non-supervised Orthologous Groups (EggNOG), NCBI Nucleotide (NT), and Kyoto Encyclopedia of Genes and Genomes (KEGG). Among these databases, NT database annotated more than 80% of acquired unigenes from each vegetable (Table S4 in Additional file 1). It indicates that around 20% of unigenes may be considered as novel transcript contigs.

### DEGs in Brassicaceae vegetables under shade

To understand how changes in the transcriptome reflect the degree of SAS in each vegetable species, DEGs were analyzed. We first checked the similarity between biological replicates per sample using Pearson’s coefficient of the normalized values. Both correlation matrix plots and principal component analysis (PCA) showed that the reproducibility of the duplicated RNA-seq samples was notably high in all vegetables (Fig. S3 in Additional file 1). These analyses also exhibited that DEGs were clearly distinguished between different time points of shade treatments (0, 1, and 6 h) (Fig. S3 in Additional file 1). Interestingly, we found different shade responses among vegetables from DEG analyses (Fig. S3b-c in Additional file 1). Both *B. rapa* Choy Sum and *B. oleracea* Kai Lan showed that DEGs from 0 and 1 h were clustered closely while the analysis in *B. rapa* Pak Choy indicated DEGs from 0 and 6 h to be one cluster (Fig. S3c in Additional file 1).

After further exclusion of low-quality transcript contigs and Trimmed Mean of M-values (TMM) normalization, a total of 32,564, 37,714, and 32,678 high-quality unigenes were obtained from Choy Sum, Kai Lan, and Pak Choy, respectively. Of these, 4064, 5074, and 5873 DEGs were evaluated to have at least 2-fold changes (≥ 2 or ≤ − 2) from Choy Sum, Kai Lan, and Pak Choy after 1 h of shade treatment, respectively (Fig. S4 in Additional file 1). As shown in “6 h vs 0 h” plots of Choy Sum and Kai Lan, the number of DEGs were clearly increased after a long shade treatment (Fig. S4 in Additional file 1). In fact, when vegetables were exposed to a longer shade treatment (6 h), the number of DEGs drastically increased up to 6402 unigenes in Choy Sum and 7922 unigenes in Kai Lan (Fig. S4 in Additional file 1). Contrastingly, 6 h shade treatment decreased the number of DEGs in Pak Choy to 5704 (Fig. S4 in Additional file 1). These results suggest that *B. rapa* Pak Choy may respond to shade slightly earlier than *B. rapa* Choy Sum and *B. oleracea* Kai Lan. Besides, the number of up- and down-regulated DEGs upon shade treatments in each vegetable were quite similar (Fig. S4 in Additional file 1). Fig. S5 (in Additional file 1) showed that the distribution of both up- and down-regulated DEGs varied in three vegetables. Based on hierarchical analysis, DEGs were classified into 8 main clusters (Fig. S5 in Additional file 1). Many genes in the cluster 2 seemed to be suppressed by shade (6 h) (Fig. S5 in Additional file 1). Functional annotation indicated that the cluster 2 genes belonged to various categories such as “very long-chain fatty acid metabolic process”, “microtubule-based movement”, and “flower development” (Supplementary Dataset 1 in Additional file 2). On the other hand, most of the genes in the cluster 3 were up-regulated in response to shade in all vegetables and the GO analysis allocated these genes into different categories such as “response to red light”, “ethylene-activated signaling pathway”, and “response to auxin” (Fig. S5 in Additional file 1 and Supplementary Dataset 1 in Additional file 2).

### Functional annotation of shade-induced DEGs from Brassicaceae vegetables

**Fig. 3:**
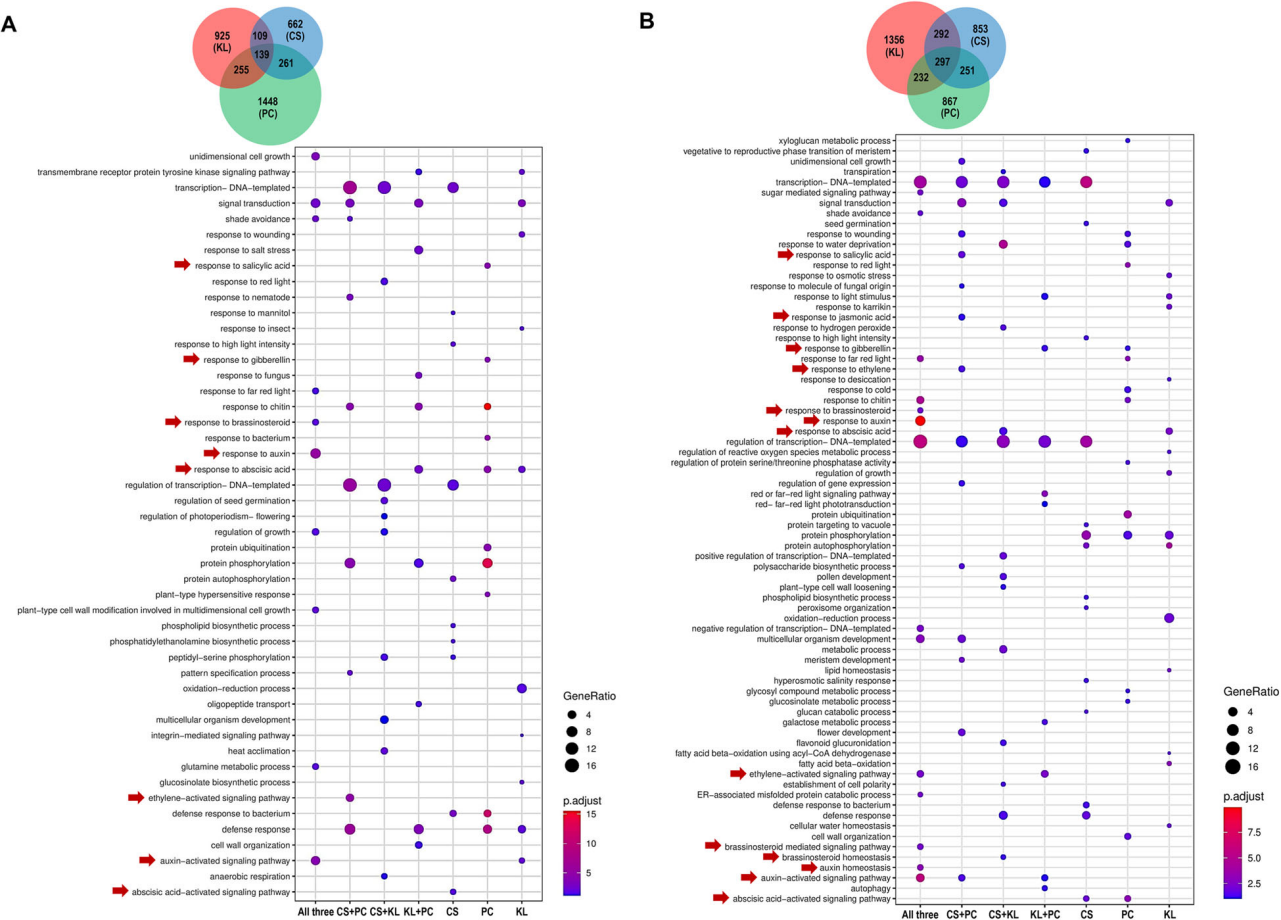
Up-regulated genes of three Brassicaceae vegetables in response to shade. Dot plots showing GO terms from up-regulated genes (1 h vs 0 h) **(a)** and (6 h vs 0 h) **(b)** of three Brassicaceae vegetables. GO categories involved in phytohormones were highlighted by red arrows. CS, Choy Sum; KL, Kai Lan; PC, Pak Choy; All three, the GO terms shared by three vegetables

Up-regulated genes under shade were analyzed and shown in Fig. 3. After merging all 1 h shade-induced genes from three vegetables, a total of 139 genes were found to be shared amongst three vegetables (Fig. 3a). The functional annotation of these 139 genes revealed various important GO categories, especially “response to auxin” and “auxin-activated signaling pathway” (Fig. 3a). Of these, *IAA19*, *IAA29*, *SMALL AUXIN UP-REGULATED RNA* (*SAURs*), *YUC8*, and *YUC9* relating to auxin signaling and biosynthesis were listed (Fig. S6a in Additional file 1 and Supplementary Dataset 2a in Additional file 3). These results exhibit that these two Brassicaceae species (all three vegetables) share a general shade response mechanism that strongly involves auxin signaling and biosynthesis (Fig. 3a). In addition, among these 139 genes, some were also found to be part of brassinosteroids (BR) signaling pathway (“response to BR”, All three, Fig. 3a). Even though all these genes were up-regulated in three vegetables grown under shade condition, the clustering analysis indicated that there were differences in gene expression between varieties (Fig. S6a in Additional file 1). Generally, there were 6 main clusters (#1 to #6, Fig. S6a in Additional file 1). Upon shade treatments, Choy Sum and Kai Lan dramatically triggered high expression in gene cluster #4 while Kai Lan and Pak Choy showed high expression in cluster #2 (Fig. S6a in Additional file 1). Specifically, *B. rapa* Pak Choy strongly induced the cluster #6 genes in comparison to the other vegetables (Fig. S6a in Additional file 1). Furthermore, transcript levels of cluster #3 genes were mostly induced in all three vegetables in response to shade condition (Fig. S6a in Additional file 1).

Apart from 139 common genes from all three vegetables, pairing of two Brassicaceae vegetables unraveled a number of DEGs that were commonly induced in response to shade (Fig. 3a). Two varieties, Choy Sum and Pak Choy, from the same species *B. rapa* showed that they both up-regulated some genes classified in “ethylene-activated signaling pathway” category under short duration of shade treatment (Fig. 3a, CS + PC). However, GO analyses of DEGs from the combinations of different Brassicaceae species, Kai Lan (*B. oleracea*) versus Choy Sum or Pak Choy (*B. rapa*) did not show any category relating to ethylene hormone (Fig. 3a, CS + KL or KL + PC). These signify that *B. rapa* varieties also activate the ethylene signaling pathway whereas *B. oleracea* vegetable mainly relies on auxin and BR to regulate very early SAS. On the other hand, each vegetable also exhibited unique GO categories in response to 1 h shade treatment. For example, *B. rapa* Choy Sum triggered “response to mannitol” genes upon shade condition while *B. rapa* Pak Choy up-regulated genes involved “response to salicylic acid (SA)” and “response to gibberellin (GA)” (Fig. 3a, CS or PC). Upon 1 h shade treatment, Kai Lan (*B. oleracea*) uniquely induced several genes (e.g. *AUXIN RESPONSE FACTOR 19*, *IAA10*, and *IAA20*) belonging to “auxin-activated signaling pathway”. Again, this result supports that *B. oleracea* Kai Lan broadly induces many genes relating to auxin to regulate SAS. As mentioned above, in addition to auxin and BR, *B. rapa* vegetables, Choy Sum and Pak Choy, triggered ethylene signaling pathway in response to short duration of shade treatment (1 h). Moreover, even though the variety-specific genes did not overlap, the GO analyses of these individual DEGs exhibit that they still function in similar signaling pathways, as seen in the prominence of abscisic acid (ABA)- and plant defense-related GO terms in each vegetable (Fig. 3a, CS, KL, or PC).

Upon longer shade treatment for 6 h, 297 genes were identified as common genes of three vegetables (Fig. 3b). The expression patterns of these 297 genes were also different between vegetables, which were able to be divided into 6 clusters (Fig. S6b in Additional file 1 and Supplementary Dataset 2b in Additional file 3). Similar to 1 h shade treatment, auxin and BR were key phytohormones responding to 6 h shade treatment in these Brassicaceae vegetables (Fig. 3b, All three). Besides, after longer exposure to shade, these vegetables also triggered the expression of various genes associated with “ethylene-activated signaling pathway” (e.g. *ETHYLENE RESPONSE DNA BINDING FACTOR 4*, *EIN3-BINDING F BOX PROTEIN 2*, and *ETHYLENE AND SALT INDUCIBLE 3*) (Fig. 3b, All three, and Supplementary Dataset 2b in Additional file 3). These data imply that the current studied Brassicaceae species employ a general shade-response mechanism mainly involving in three phytohormones, auxin, BR, and ethylene. Moreover, we found that auxin and BR mainly contribute to SAS from early stage (1 h) while ethylene participates in this process in the later stage (6 h).

With regards to other variety-specific DEGs, each vegetable continued to induce ABA-related genes in a variety-specific manner (Fig. 3b). Other phytohormone-related genes were found to be shared between two *B. rapa* vegetables, Choy Sum and Pak Choy, having a conserved induction of auxin, ethylene, jasmonic acid (JA) and SA-related genes (Fig. 3b, CS + PC). In contrast, comparison between two species, *B. oleracea* (Kai Lan) and *B. rapa* (Pak Choy), unraveled that they shared up-regulated DEGs involved in GA and ethylene (Fig. 3b, KL + PC). Instead, DEGs involved in “flower development” and “meristem development” were particularly shared in *B. rapa* vegetables, Choy Sum and Pak Choy (Fig. 3b, CS + PC). Conversely, *B. oleracea* Kai Lan up-regulated the expression of genes related to the “regulation of growth” at 6 h shade (Fig. 3b, KL). These results suggest that at longer duration of shade, transcriptional regulation in these two Brassicaceae species (all three vegetables) starts to change from phytohormone-specific to focus on developmental alterations (Fig. 3b).

### Functional annotation of down-regulated DEGs in Brassicaceae vegetables under shade

**Fig. 4:**
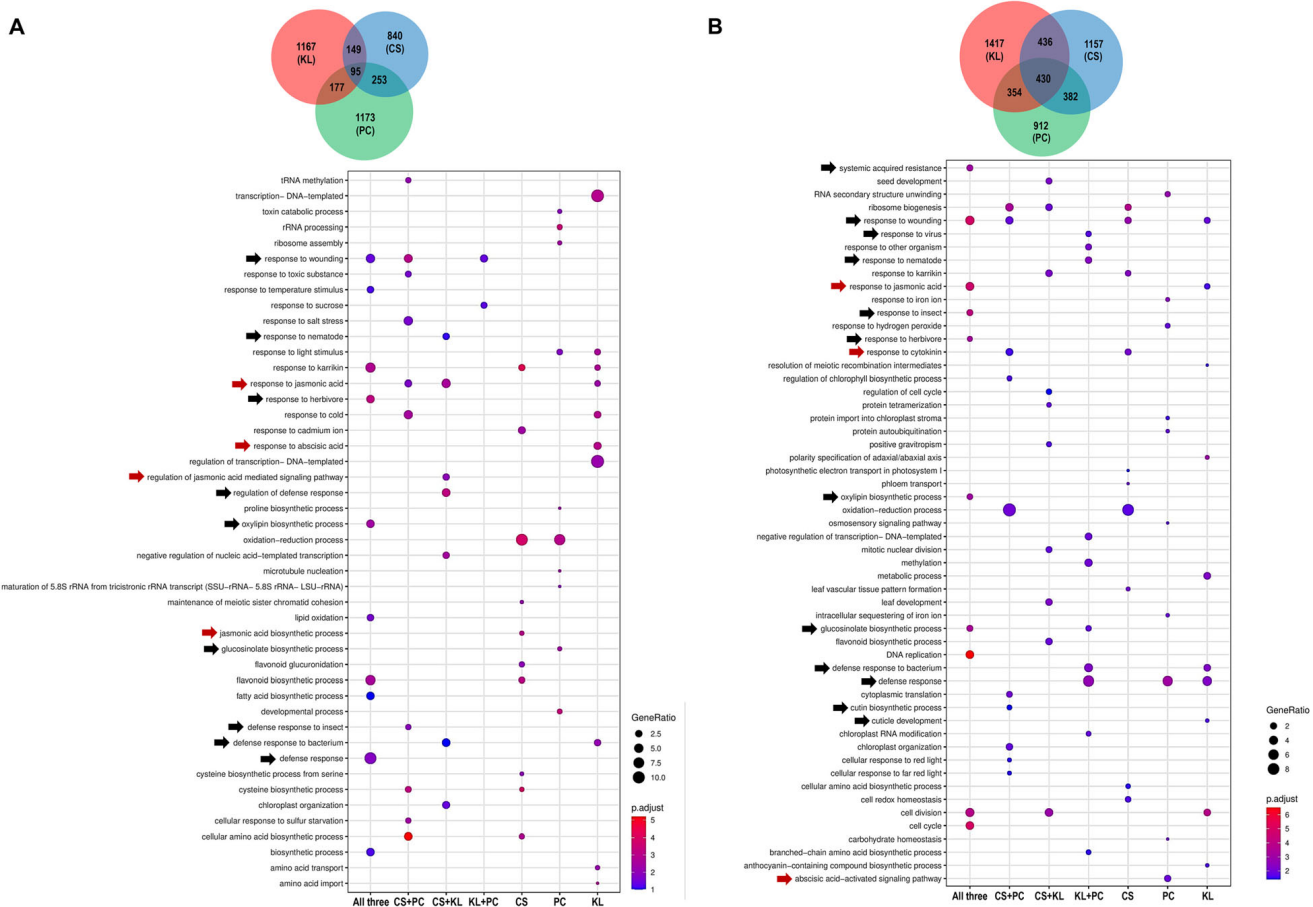
Down-regulated genes of three Brassicaceae vegetables in response to shade. Dot plots showing GO terms from down-regulated genes (1 h vs 0 h) **(a)** and (6 h vs 0 h) **(b)** of three Brassicaceae vegetables. GO categories involved in phytohormones or plant defenses were highlighted by red or black arrows, respectively. CS, Choy Sum; KL, Kai Lan; PC, Pak Choy; All three, the GO terms shared by three vegetables

Next, we investigated down-regulated genes under shade from all Brassicaceae vegetables. After merging these down-regulated genes from three Brassicaceae vegetables, 95 and 430 genes were sorted out as common genes at 1 and 6 h shade treatment, respectively (Fig. 4 and Supplementary Dataset 3 in Additional file 4). Common GO categories such as “response to herbivore”, “defense response”, “flavonoid biosynthetic process”, and “oxylipin biosynthetic process” were highly counted from the 95 genes batch (Fig. 4a, All three). Based on the clustering analysis of shade-down-regulated DEGs, we found that Choy Sum and Pak Choy as varieties of *B. rapa* exhibited a similar pattern while Kai Lan (*B. oleracea*) showed a different one (Fig. S7a in Additional file 1). For instance, both *B. rapa* vegetables slowly reduced the expression of genes belonging to cluster #1, whereas *B. oleracea* (Kai Lan) had comparatively lower expression of genes in this cluster (Fig. S7a in Additional file 1). On the other hand, *B. rapa* vegetables genes in cluster #3 were repressed drastically while the same cluster genes in *B. oleracea* (Kai Lan) were expressed at relatively higher levels and gradually down-regulated in response to shade (Fig. S7a in Additional file 1). The functional annotation of 430 genes obtained from 6 h batch revealed that a longer shade treatment repressed the expression of genes involved in “response to wounding” (e.g. *MYB29*, *LIPOXYGENASE 2*, and *LIPOXYGENASE 4*), “response to insect” (e.g. *KUNITZ-PROTEASE INHIBITOR 1* and *ARABIDOPSIS PHYTOALEXIN DEFICIENT 4*), “response to herbivore” (e.g. *HEVEIN-LIKE*), “response to JA” (e.g. *JASMONATE-ZIM-DOMAIN PROTEIN 9* and *JASMONIC ACID RESPONSIVE 2*) as well as “glucosinolate biosynthetic process” (Fig. 4b and Supplementary Dataset 3b in Additional file 4). Furthermore, GO analysis of species-specific down-regulated genes were also unraveled. As shown in Fig. 4b, in response to 6 h shade treatment, *B. rapa* vegetables, Choy Sum and Pak Choy, reduced the transcript levels of genes belonging to “response to cytokinin” while *B. oleracea* Kai Lan down-regulated genes involving in “polarity specification of adaxial/abaxial axis” and “cuticle development”.

### Shade-induced auxin biosynthesis in Brassicaceae vegetables

**Fig. 5:**
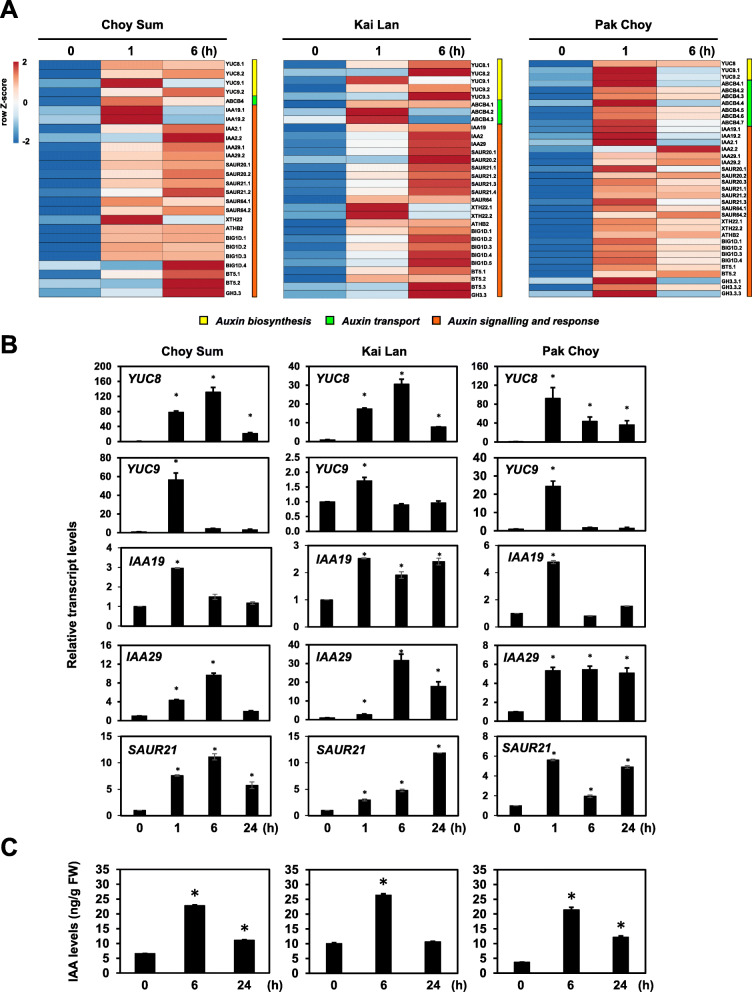
Auxin as a key hormone regulating SAS in three Brassicaceae vegetables. **a** Representative auxin-related genes which were up-regulated in response to shade treatments (0, 1, and 6 h) obtained from RNA-seq data from three Brassicaceae vegetables were shown in heat-maps. Scale bar color corresponds to row Z-score. *ABCB4*, *ATP-binding cassette B4*; *XTH22*, *Xyloglucan endotransglucosylase/hydrolase protein 22*; *ATHB2*, *Arabidopsis thaliana homeobox protein 2*; *BIG1D*, *Big Grain 1*; *BT5*, *BTB and TAZ domain protein 5*; *GH3.3*, *auxin-responsive GH3-like protein 3*. **b** qRT-PCR analysis of auxin-related genes in two-week-old vegetables, Choy Sum, Kai Lan, and Pak Choy treated with shade for 1, 6, and 24 h. The qRT-PCR signals were normalized to internal control (*BraACTIN2*). Bars represent average ± SD. n = 3. Columns marked with an asterisk indicate significantly different from Control (0 h) (*p-value* < 0.05). **c** Quantification of IAA in three Brassicaceae vegetables upon shade treatment for 6 and 24 h. Bars represent average ± SD. n = 3. Columns marked with an asterisk indicate significantly different from Control (0 h) (*p-value* < 0.05)

As shown in Fig. 5a, RNA-seq data revealed that shade notably induced the mRNA levels of genes involved in auxin biosynthesis (*YUC8* and *9*), transport (*ATP-binding cassette B4*), and signaling in all three vegetables. In Arabidopsis, four among 11 *YUC* genes, namely *YUC2*, *5*, *8*, and *9*, were found to be induced by shade treatment [23]. Here, two homologs of *YUC8* and *9* were commonly induced by shade in all three vegetables (Fig. 5a). In addition, numerous auxin signaling genes such as *IAA19*. *IAA29*, *SAUR20*, and *SAUR21* were also revealed to be activated upon shade in all these Brassicaceae species (Fig. 5a). To further validate our RNA-seq results, we verified the gene expression of selected shade-induced genes (*YUC8*, *YUC9*, *IAA19*, *IAA29*, and *SAUR21*) using quantitative reverse transcription polymerase chain reaction (qRT-PCR). Consistent with the RNA-seq data, all selected genes from all present Brassicaceae species were significantly induced by shade treatments for 1, 6, and 24 h (Fig. 5b). Generally, the expression of these genes strongly increased after 1 or 6 h shade-exposure and transcript levels were decreased subsequently at 24 h shade treatment (Fig. 5b). Overall, these Brassicaceae species triggered their transcriptional responses very early after exposure to shade and these alterations in gene expression led to changes in their growth and development as SAS.

Many lines of evidence have shown that shade-induced elongation of hypocotyl and leaf petiole in Arabidopsis is clearly involved in the increase of endogenous auxin levels [13, 23]. In Brassicaceae vegetables, our RNA-seq and qRT-PCR analyses also indicated that auxin biosynthetic genes such as *YUC8* and *YUC9* were significantly induced in response to shade (Supplementary Dataset 2 in Additional file 3 and Fig. 5a-b). Therefore, we measured endogenous levels of indole-3-acetic acid (IAA) as the most abundant natural auxin in plants (Fig. 5c). Consistent with RNA-seq and qRT-PCR data, IAA levels in shoot tissues were found to peak at 6 h but decreased by 24 h shade treatment (Fig. 5c). Besides, IAA levels decreased back to baseline in *B. oleracea* Kai Lan at 24 h shade treatment, but not for *B. rapa* vegetables, Choy Sum and Pak Choy (Fig. 5c). Consistent with findings in Arabidopsis, this result supports that auxin plays a key role in shade response in the Brassicaceae species and that early increment of auxin biosynthesis is required to promote the elongation of hypocotyl and leaf petiole.

**Fig. 6:**
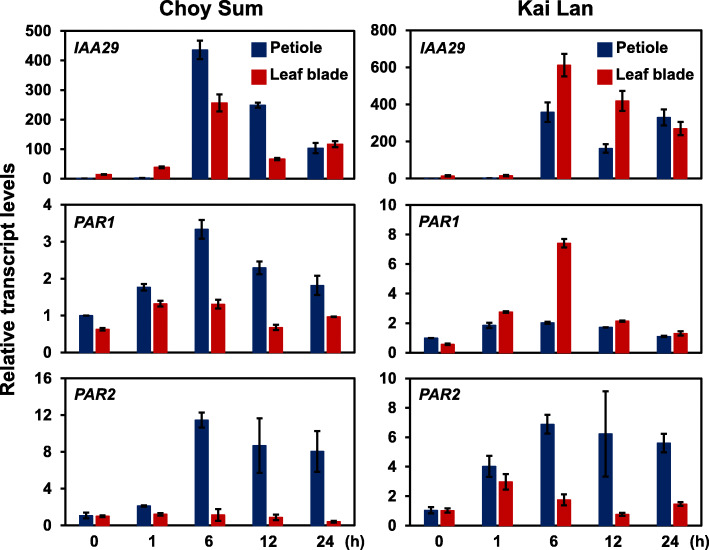
Transcript levels of shade-responsive genes in leaf blade and petiole of Choy Sum and Kai Lan. qRT-PCR analysis of shade-responsive genes in leaf blade and petiole of two-week-old vegetables, Choy Sum and Kai Lan treated with shade for 1, 6, 12, and 24 h. The qRT-PCR signals were normalized to internal control (*BraACTIN2*). Bars represent average ± SD. n = 3

Under shade condition, *B. oleracea* Kai Lan significantly elongated its leaf petiole whereas the other two *B. rapa* vegetables, Choy Sum and Pak Choy exhibited this phenotype at moderate and minor levels, respectively (Fig. 1b). In addition, the present transcriptomic analysis indicated that shade signal transduction in *B. oleracea* Kai Lan somewhat differs from *B. rapa* vegetables, Choy Sum and Pak Choy. To further explore the differences in shade response at the tissue level, we examined the expression of representative shade-responsive genes in leaf blade and petiole of these two species, *B. oleracea* Kai Lan and *B. rapa* Choy Sum (Fig. 6). Basically, shade successfully induced transcript levels of shade-responsive genes, *IAA29*, *PHY RAPIDLY REGULATED 1* (*PAR1*), and *PAR2* in leaf blade and petiole of both species, *B. oleracea* Kai Lan and *B. rapa* Choy Sum (Fig. 6). However, the tissue-specific expression of these genes varied (Fig. 6). *IAA29* and *PAR1* were induced stronger in Choy Sum petiole than leaf blade while Kai Lan exhibited an opposite pattern (Fig. 6). In Kai Lan petiole, fold changes of *IAA29* and *PAR1* were similar to those of Choy Sum. However, Kai Lan leaf blade has a stronger shade-induction of *IAA29* and *PAR1* than Choy Sum (Fig. 6). On the other hand, the induction of *PAR2* was found to be higher in both Choy Sum and Kai Lan petiole than their leaf blade (Fig. 6). Thus, at the tissue level, each Brassicaceae species may specifically trigger different shade-responsive genes and these lead to species-specific SAS phenotypes.

### Shade-repressed genes for anthocyanins biosynthesis and chemical defensive compounds in Brassicaceae vegetables

**Fig. 7:**
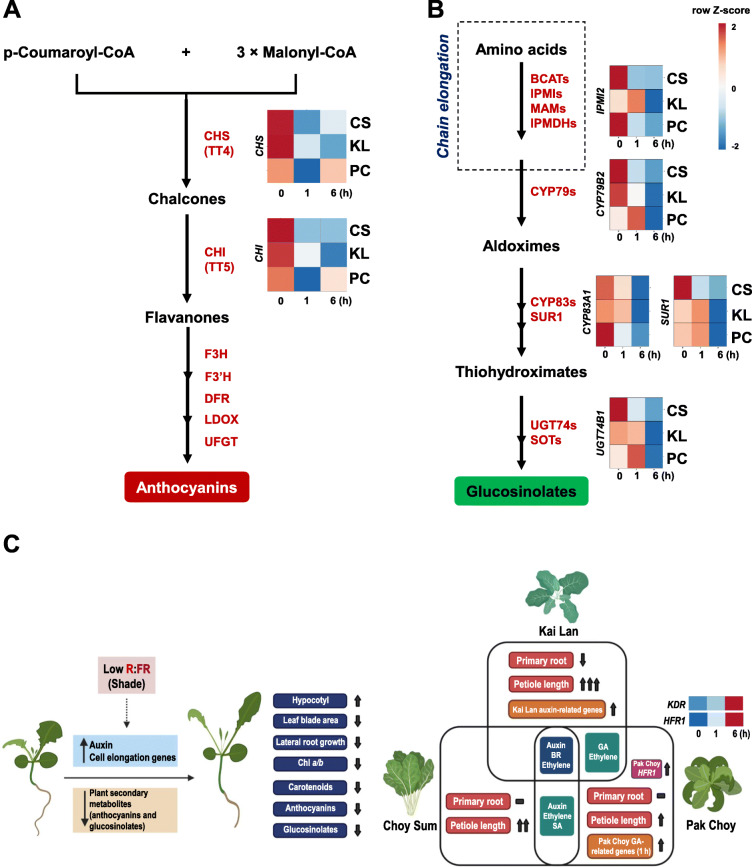
Biosynthesis of anthocyanins and glucosinolates in three Brassicaceae vegetables under shade. **a** Schematic pathway of anthocyanins biosynthesis and the expression of genes (*CHS* and *CHI*) involved in this pathway in response to shade treatments (1 and 6 h). **b** Schematic pathway of glucosinolates biosynthesis and the expression of genes (*IPMI2*, *CYP79B2*, *CYP83A1*, *SUR1*, and *UGT74B1*) involved in this pathway in response to shade treatments (1 and 6 h). CS, Choy Sum; KL, Kai Lan; PC, Pak Choy; *CHS*, *chalcone synthase*; *CHI*, *chalcone isomerase*; *IPMI2*, *isopropylmalate isomerase 2*; *CYP79B2*, *cytochrome p450 79B2*; *CYP83A1, cytochrome p450 83A1*; *SUR1*, *superroot 1*; *UGT74B1*, *UDP-glucosyltransferase 74B1*. **c** A graphical overview indicates the general and distinguished SAS in three Brassicaceae vegetables. This highlights that auxin, BR, and ethylene function as common SAS hormones in these vegetables. In addition, Kai Lan also activates its unique auxin-related genes while Pak Choy quickly induces GA-signaling genes in response to shade. *HFR1*, *LONG HYPOCOTYL IN FAR-RED 1*; *KDR*, *KIDARI*. Scale bar color corresponds to row Z-score

Anthocyanins is one of well-known flavonoid groups in plants. As indicated in Fig. 2, shade drastically reduced the accumulation of total anthocyanins in both two Brassicaceae species (all three vegetables). Two representative genes, *chalcone synthase* (*CHS*) and *chalcone isomerase* (*CHI*), encoding for enzymes that catalyze the rate-limiting steps of anthocyanins biosynthesis pathway were clearly suppressed by shade treatments (Fig. 7a). Glucosinolates, sulfur-containing compounds mainly found in Brassicaceae plants have been considered to play a role in plant defense against pathogens [22]. In Arabidopsis, glucosinolates are synthesized from amino acids through several steps using branched-chain amino acid aminotransferase (BCAT), methylthioalkylmalate synthase (MAM), isopropylmalate isomerase (IPMI), isopropylmalate dehydrogenase (IPMDH), cytochromes P450 (CYP79s and CYP83s), *C-S* lyase (SUPERROOT 1, SUR1), UDP-glucose:thiohydroximate *S*-glucosyltransferase (UGT74s), and sulfotransferase (SOTs) (Fig. 7b and [24]). Our RNA-seq of Brassicaceae vegetables revealed that shade repressed the expression of *IPMI2*, *CYP79B2*, *CYP83A1*, *SUR1*, and *UGT74B1* encoding for the glucosinolates biosynthetic enzymes (Fig. 7b).

## Discussion

### Conserved mechanism underlying SAS in Brassicaceae vegetables

In this study, the RNA-seq and de novo transcriptome assembly were employed to firstly investigate underlying mechanisms of SAS in two different Brassicaceae species, *B. rapa* (Choy Sum and Pak Choy) and *B. oleracea* (Kai Lan). Phenotypic characterizations showed that shade induced hypocotyl elongation, increased hypocotyl cell size, and reduced root growth in all two Brassicaceae species (Fig. 1a and Fig. S1–2 in Additional file 1) that are similar to those in Arabidopsis plants [2]. In addition, fresh weight was significantly reduced due to decreased leaf blade area when the plants were grown under simulated shade condition (Fig. 1b). These data exhibit that shade negatively influences the growth performance leading to reduction of productivity of all two Brassicaceae species. A previous study in a conifer species also found that the Norway spruce seedlings reduced their biomass under shade, which was proposed to optimize the light capture in this conifer species [18]. It is plausible to have a conserved SAS mechanism in both angiosperms and gymnosperms regarding the biomass production upon shade condition.

Transcriptome analysis revealed that almost 20% of obtained unigenes from each vegetable were not matched to any available database, implying that these may be novel transcript contigs which have not been reported previously (Table S4 in Additional file 1). The functional annotation of the shade-induced genes in all three vegetables identified several phytohormones such as auxin, BR, and ethylene, all of which might be responsible for the SAS of these vegetables (Fig. 1 and Fig. 3). Transcriptome analyses of Arabidopsis seedlings exposed to simulated shade (low R/FR ratio and low PAR) showed that many of genes involved in the biosynthesis and signaling of auxin and BR were induced under shade [13, 14, 25]. These results support our RNA-seq data and analysis from simulated shade-treated vegetables, as they are well consistent with previous studies in Arabidopsis. Auxin is a key phytohormone regulating the SAS in Arabidopsis [4, 13, 14]. Under shade condition, auxin biosynthesis, polar transport, and sensitivity are briskly activated, and this subsequently stimulates the cell elongation in plant tissues including hypocotyl and leaf petiole [4]. We also found a number of auxin-related genes including biosynthesis, transport, and signaling (e.g. *YUC8*, *YUC9*, *IAA19*, *IAA29*, and *SAURs*) that were highly induced by shade in all three Brassicaceae vegetables (Supplementary Dataset 2a in Additional file 3 and Fig. 5a-b). In Arabidopsis, YUCs were well characterized as the critical enzymes converting indole-3-pyruvic acid to IAA in the tryptophan-dependent auxin biosynthesis pathway [23]. The expression of several Arabidopsis *YUCs* (e.g. *YUC2*, *5*, *8*, and *9*) were found to be highly up-regulated by shade treatment [23]. In this study, as unraveled by the RNA-seq and qRT-PCR, Brassicaceae vegetables *YUC8* and *9* homolog genes were also activated at very early stages of shade exposure (1 and 6 h) and the induction was attenuated in response to a longer duration (24 h) (Fig. 5a-b). Indeed, auxin levels were swiftly increased in vegetables when they were exposed to shade (Fig. 5c). This is consistent with previous findings that the shade signal (low R/FR ratios) promptly increased auxin levels in Arabidopsis, and mutants relating to auxin biosynthesis such as *sav3/taa1* (*SHADE AVOIDANCE3*/*TAA1*) and *yuc2589* failed to elongate the hypocotyl upon shade treatment [13, 23]. Overall, the gene expression analyses and IAA quantification data indicate that both *B. oleracea* (Kai Lan) and *B. rapa* (Choy Sum and Pak Choy) employ the rapid biosynthesis of auxin, so that this hormone can activate the down-stream signaling pathways to adjust their growth performance under shade. In addition to the key SAS hormone, auxin, other phytohormones such as GA, ethylene, and BR have been documented to be involved in the regulation of SAS [26]. Upon shade treatment, Brassicaceae vegetables also induced the transcript levels of the genes involved in BR and ethylene. Altogether, our results unravel a conserved shade response mechanism which involves three SAS hormones, auxin, BR, and ethylene, in Brassicaceae vegetables.

### Variety-specific SAS in Brassicaceae species

In addition to common SAS, each vegetable also exhibited its distinctive phenotypes and GO categories in response to shade (Fig. 1 and Fig. 3). Kai Lan (*B. oleracea*) dramatically impaired primary root length and elongated leaf petiole upon shade condition (Fig. 1). On the other hand, both Choy Sum and Pak Choy (*B. rapa*) seemed to not reduce primary root growth and slightly increased leaf petiole length when they were grown under shade condition (Fig. 1). A prior study showed that low R/FR ratio might reduce the auxin transport or response in the root tissue, and this subsequently impaired the lateral root growth [27]. Furthermore, ELONGATED HYPOCOTYL 5 (HY5) transcription factor was found to move from shoot to root and inhibit the auxin signaling and transport, which negatively influence the lateral root growth [28, 29]. As mentioned earlier, the *B. oleracea* Kai Lan shoots accumulated a higher amount of auxin (IAA) than those in *B. rapa* vegetables, Choy Sum and Pak Choy, under shade (Fig. 5c). This implies that *B. oleracea* Kai Lan may prefer to retain IAA in the shoot tissue and decrease auxin polar transport to the roots, thus resulting in less root growth.

Based on the RNA-seq analysis, each vegetable also exhibited its particular GO categories in response to shade treatment. For instance, *B. rapa* vegetables, Choy Sum and Pak Choy triggered genes involving in “ABA-activated signaling pathway” and “response to GA”, respectively (Fig. 3a). On the other hand, *B. oleracea* Kai Lan induced genes belonging to “response to wounding” and “auxin-activated signaling pathway” upon 1 h shade treatment. It is worth noting that these *B. oleracea* (Kai Lan) genes categorized in “auxin-activated signaling pathway” did not overlap with other auxin-related genes in *B. rapa* vegetables, Choy Sum and Pak Choy. This suggests that *B. oleracea* Kai Lan widely activates a larger number of genes involving in auxin to regulate its SAS. Furthermore, as indicated in Fig. 5a, most of the auxin signaling genes in *B. oleracea* Kai Lan were gradually induced when the plants were exposed to prolonged shade duration (6 h versus 1 h). In contrast, the transcript levels of these genes in *B. rapa* Pak Choy were peaked at 1 h shade treatment (Fig. 5a). On the other hand, another *B. rapa* Choy Sum did not show a clear trend for these gene expressions as compared to other vegetables (Fig. 5a). These results suggest that each Brassicaceae species may possess a specific auxin signal transduction mechanism in response to shade condition. These data support the severe SAS in *B. oleracea* Kai Lan in comparison to other *B. rapa* vegetables.

In agreement with a previous study in Arabidopsis [30], shade also induced the transcript levels of *PAR1* and *PAR2* in *B. rapa* Choy Sum and *B. oleracea* Kai Lan (Fig. 6). However, expression levels of these shade-induced genes varied between leaf blade and petiole of these Brassicaceae species (Fig. 6). For instance, the transcript level of *B. rapa* Choy Sum *PAR1* was more than 3 times at 6 h shade treatment when compared with control (0 h) while the *B. oleracea* Kai Lan *PAR1* was only increased to around 2 times in leaf petiole tissues (Fig. 6). In the case of *PAR2* homologs, at the same tissue (leaf petiole), shade-induced expression was also higher in *B. rapa* Choy Sum (~ 12 times) than *B. oleracea* Kai Lan (~ 7 times). As two basic helix-loop-helix (bHLH)-like proteins, PAR1 and PAR2 function as negative regulators of SAS in Arabidopsis [31]. The different expression levels of vegetable *PAR* genes may also associate with or even reflect petiole elongation rates in the two varieties under shade (Fig. 1b). These imply that the underlying regulatory mechanisms of gene expression upon shade greatly vary between different Brassicaceae species.

The bHLH gene family was comparatively analyzed in several Brassicaceae species, *B. oleracea*, *B. rapa*, and *B. napus* [32]. In Arabidopsis, AtHFR1, an atypical member of the bHLH family that lacks the DNA-binding capacity, was showed to negatively regulate the SAS via interacting PIF4/5 and thereby prohibiting the functions of these transcription factors [33]. Recently, an ortholog of *AtHFR1* was also found to suppress the shade response in another Brassicaceae species, *Cardamine hirsuta* [34]. Here, the RNA-seq data revealed that only Pak Choy induced the expression of *HFR1* gene in response to shade treatment (Fig. 7c). Moreover, Pak Choy was also shown as the most tolerant variety among three tested vegetables to shade (Fig. 1). These data imply that Pak Choy *HFR1* may also function as a negative regulator of SAS in this vegetable. Another non-DNA-binding HLH protein, KIDARI (KDR) was reported in regulating shade avoidance network through interaction with a number of negative regulators of SAS in Arabidopsis [30]. This study proposed that HFR1 and KDR may act independently in the regulation of hypocotyl elongation in response to shade [30]. Interestingly, the present RNA-seq of three Brassicaceae varieties indicated that only *B. rapa* Pak Choy *KDR* gene was induced under shade (Fig. 7c). The induction of *HFR1* and *KDR* in *B. rapa* Pak Choy by shade suggests that these two proteins may both function as SAS regulators in this vegetable. It would be interesting to further study the regulatory pathways of vegetable SAS which may be largely controlled by various vegetables homologs of Arabidopsis HFR1, KDR, PAR1, PAR2, and other bHLH factors.

### Trade-offs between plant growth and defense under shade

Besides up-regulated genes, shade also down-regulated a large number of genes in Brassicaceae vegetables (Fig. S4 and Fig. S7 in Additional file 1). In general, shade signals decrease plant defense and sensitivity to JA, one of key phytohormones mediating plant defense responses [35–37]. Low R/FR ratios reduced JA sensitivity through inactivation of phyB in Arabidopsis [35, 36]. In corroboration with Arabidopsis studies on trade-offs between plant growth and defense [35–37], our RNA-seq data and GO analysis unveiled that these shade-exposed Brassicaceae vegetables repressed expression of genes that function in plant defense against bacteria and insects, JA biosynthetic, and JA signaling pathways (Fig. 4). These results suggest that the mechanism underlying trade-offs between plant growth and defense under shade might be conserved between Brassicaceae vegetables and Arabidopsis.

Glucosinolates which are mainly present in the members of family Brassicaceae have been implicated in plant defense against pathogens and as beneficial for human health by having anti-inflammation and anti-cancer properties [22, 24, 38, 39]. Genes such as *IPMI2*, *CYP79B2*, *CYP83A1*, *SUR1*, and *UGT74B1* for the biosynthesis of glucosinolates were down-regulated in response to shade treatment, especially after 6 h treatment (Fig. 7b). Biosynthesis of indole glucosinolate and auxin IAA are diverged at indole-3-acetaldoxime [40]. This implies that Brassicaceae plants may reduce the biosynthesis of the glucosinolate to redirect the metabolic flux toward auxin accumulation in response to shade.

## Conclusion

In this study, auxin was shown to function as a key SAS phytohormone which regulated the growth of both *B. rapa* (Choy Sum and Pak Choy) and *B. oleracea* (Kai Lan) under shade condition. On the other hand, each variety or species of Brassicaceae vegetables also responded distinctively to shade treatment at both molecular and phenotypic levels. For instance, *B. rapa* Pak Choy quickly activated GA-related genes upon 1 h shade treatment whereas *B. oleracea* Kai Lan seemed to rely more on the auxin signaling pathway for SAS. Based on phenotypic and transcriptomic analyses, *B. oleracea* Kai Lan was found to be very susceptible while *B. rapa* Pak Choy was relatively tolerant to shade. Importantly, this study generated a genome-wide transcriptomic information regarding SAS in different Brassicaceae varieties. Besides, two different *B. rapa* vegetables, Choy Sum and Pak Choy, also showed different SAS levels under shade. This suggests that each variety, even from a same species, may specifically require an optimal light-growth condition. Altogether, these data are useful for further studies on SAS mechanisms and can be also used in translational research aiming to improve the productivity of vegetables grown in high-density conditions such as indoor vertical farming.

## Methods

### Plant materials and growth conditions

Three Brassicaceae vegetables, Choy Sum (*B. rapa* var. *parachinensis*), Kai Lan (*B. oleracea var. alboglabra*), and Baby White Pak Choy (*B. rapa* subsp. *Chinensis*) were used in this study. Brassicaceae vegetable seeds were surface sterilized with bleach solution (NaClO 1%), washed 5 times with sterile water, and stored at 4 °C for 3 days. Subsequently, sterile seeds were placed on Murashige and Skoog (MS) medium for 4 days and grown in a growth chamber at 21 ± 1 °C, 16 h light/8 h dark cycle, relative humidity of approximately 60%, and with a normal light condition (LED light, PAR = ~ 100 μmol photons m^− 2^ s^− 1^; red = 5 μmol photons m^− 2^ s^− 1^; far-red = 1 μmol photons m^− 2^ s^− 1^). Whole seedlings of 4-day-old vegetables were used for simulated shade treatment as follows, PAR = ~ 15 μmol photons m^− 2^ s^− 1^, red = 1 μmol photons m^− 2^ s^− 1^, far-red = 8 μmol photons m^− 2^ s^− 1^ (16 h light/8 h dark cycle). After 1 week, vegetables were used for measuring lengths of hypocotyls and primary roots.

Two-week-old vegetables grown on soil were treated with shade for 0, 1, 6, 12 and 24 h. The shoots were used for RNA-seq, qRT-PCR, and IAA quantification. Similarly, two-week-old vegetables grown on soil were treated with shade for 1 week to examine SAS phenotypes.

### RNA extraction, library construction, and RNA-seq

Aerial parts of vegetables were homogenized in liquid nitrogen and used for total RNA extraction using Ribospin™ Plant kit (GeneAll Biotechnology, Seoul, South Korea). The RNA quality and quantity were checked by Agilent Technologies 2100 Bioanalyzer (Agilent Technologies, Santa Clara, CA, United States). RNA samples which have an RNA Integrity Number (RIN) value greater than 7 were used for TruSeq stranded mRNA library construction (Illumina, San Diego, CA, United States). The paired-end sequencing was performed by NovaSeq 6000 system (Illumina, San Diego, CA, United States) at Macrogen Asia Pacific Pte. Ltd. (Singapore).

### Quality control of RNA-seq and de novo transcriptome assembly

After RNA-seq, the raw data were processed with Trimmomatic program (version 0.38) to remove adapter sequences [41]. Next, the quality of produced data was determined by the Phred quality score at each cycle using FastQC (version 0.11.7) with default parameters (http://www.bioinformatics.babraham.ac.uk/projects/fastqc). The total number of read bases and reads as well as GC percentage and Phred quality score (Q20 and Q30) were calculated and shown in Table S1 (in Additional file 1). The trimmed data of each sample was then merged into one file to construct combined reference and the de novo transcriptome assembly was conducted by Trinity using default parameters (version trinityrnaseq_r20140717) [42].

### Gene functional annotation

Assembled contigs were clustered into the non-redundant (nr) transcript contigs to be “unigenes” using CD-HIT-EST program (version 4.6) [43]. Then, unigenes were used for predicting the open reading frames (ORFs), functional annotation, and DEGs analysis.

For functional annotation, unigenes were searched against different public databases including Gene Ontology (GO, http://www.geneontology.org/, version v20180319), UniProt (http://www.uniprot.org/, version 20,180,116), National Center for Biotechnology Information (NCBI) nr protein database (https://www.ncbi.nlm.nih.gov/protein/, version 20,180,503), Pfam protein families database (https://pfam.xfam.org/, version 20,160,316), evolutionary genealogy of genes: Non-supervised Orthologous Groups (EggNOG, http://eggnogdb.embl.de/, version eggnog4), NCBI Nucleotide (https://www.ncbi.nlm.nih.gov/nucleotide/, version 20,180,116), and Kyoto Encyclopedia of Genes and Genomes (KEGG, http://www.genome.jp/kegg/ko.html, version 20,190,104).

### DEGs analysis

DEGs analysis was carried out based on read-count for unigenes obtained through the RSEM program (version v1.3.1) with default parameters [44]. During data pre-processing, if more than one read-count value was 0, it was excluded from the analysis. Next, to estimate relative RNA production levels, standard TMM normalization was performed in edgeR (version 3.22.5; R version 3.5.0). Statistical analysis of DEGs was performed using Fold Change, exactTest using edgeR per comparison pair. Statistically significant DEGs were then selected on conditions of Fold Change ≥2 or ≤ − 2 and adjusted *p-value* < 0.05.

To deeply annotate the functions of shade-responsive DEGs in Brassicaceae vegetables, all DEGs were processed against the Arabidopsis database (The Arabidopsis Information Resource) to identify the Arabidopsis homologs and corresponding Arabidopsis Gene IDs. Further GO analyses were performed using these Arabidopsis Gene IDs in the Database for Annotation, Visualization, and Integrated Discovery (DAVID version 6.8, https://david.ncifcrf.gov). Top 10 significantly enriched functional groups were selected and shown.

For hierarchical clustering analyses showing in heat-maps, Euclidean Method and Complete Linkage were used to group the similarity of contigs and samples by expression level (normalized value) from significant list more than at least one of total comparison pairs.

### qRT-PCR

Total RNA was used for first-strand cDNA synthesis by M-MLV Reverse Transcriptase (Promega, Wisconsin, United States). qPCR was carried out using TB Green Premix Ex Taq (Takara Bio, Shiga, Japan) with specific primers listed in Table S5 (in Additional file 1). A homologous gene (*BraACTIN2*) of *Arabidopsis ACTIN2* from each vegetable was included as the internal control. The thermocycling was (95 °C for 3 min) × 1 cycle and (95 °C for 15 s and 60 °C for 30 s) × 40 cycles. The relative transcript level was determined by the 2^-∆∆Ct^ method [45], where ∆Ct = Ct^*Gene*^ - Ct^*BraACTIN2*^ and ∆∆Ct = ∆Ct^1-h or 6-h^ - ∆Ct^0-h^.

### Quantification of Chl *a*/*b*, total carotenoids, and total anthocyanins

Four-day-old or 2-week-old vegetables grown under normal light or shade conditions for 1 week were used for quantification of Chl *a*/*b*, total carotenoids, and total anthocyanins. Vegetable samples were ground in liquid nitrogen to obtain the fine powder. Approximately 100 mg (fresh weight) of the plant powder was re-suspended in 1 ml of absolute methanol and kept on ice in darkness for 20 min. After centrifugation at 16,000 *g* at 4 °C for 4 min, the supernatant of each sample was transferred to a new tube. The pellet was re-extracted using methanol until they lose all coloration and all supernatants of each sample were pooled into one tube. A spectrometer (Spark multimode microplate reader, Tecan, Switzerland) was used to measure the absorbance values at 470 nm, 653 nm, and 666 nm to estimate the concentration of Chl *a*, Chl *b* and total carotenoids, based on the formulas as described elsewhere [46].

For total anthocyanins quantification, 300 μl of absolute methanol (supplemented with 1% HCl v/v) was added to each 100 mg (fresh weight) of plant powder. After rigorous vortexing, samples were stored overnight at 4 °C in darkness. Next, same volume of distilled water and chloroform was added to each sample. After rigorous vortexing, the samples were centrifuged at 16,000 *g* at 22 °C for 3 min. The supernatant (100 μl/ each) was subsequently transferred to a well of 96-well microplate to determine the absorbance values at 535 and 650 nm by Spark multimode microplate reader (Tecan, Switzerland). Total anthocyanins level was calculated by the following formula: [(A_535_ – A_650_)/ g fresh weight] [47].

### Quantification of IAA by ultra-high-performance liquid chromatography tandem mass spectrometry (UHPLC-MS/MS)

Powdered samples from 100 mg of each vegetable were extracted with methanol (80%) for 30 min at room-temperature. Samples were then centrifuged to remove the cell debris. Each supernatant was concentrated in a vacuum concentrator and used for IAA measurement by a UHPLC-MS/MS system in negative ionization mode (Thermo Fisher Scientific, Massachusetts, United States). UHPLC conditions were described elsewhere [48]. Basically, liquid chromatography separation was carried out using Accucore RP-MS column (Thermo Fisher Scientific, Massachusetts, United States) with 5 mM acetic acid in 5% (v/v) acetonitrile as mobile phase A, 5 mM acetic acid in 95% (v/v) acetonitrile as mobile phase B, and the following run conditions: 5% B at 0–3 min, gradient of 5 to 95% B at 3–6 min, holding at 95% B at 6–10 min, followed by gradient of 95 to 5% B at 10–10.1 mins, and re-equilibration at 5% B at 10.1–11 min. The injection volume for all samples was 5 μl and flow rate was fixed at 0.3 ml min^− 1^ throughout. Analytical standard for IAA was purchased (Sigma-Aldrich, Missouri, United States) and used for preparation of the standard curve. Data analysis was done using TraceFinder 4.1 software (Thermo Fisher Scientific, Massachusetts, United States).

### Statistical analysis

The shade treatment experiments, qRT-PCR, and IAA quantification were repeated independently three times at least. The statistical analysis was performed using independent two-sample *t*-test (*p-value < 0.05)* with the followed formula.

$$ t=\frac{Mean_{Control}-{Mean}_{Treatment}}{S_p\sqrt{\frac{2}{n}}} $$

In this formula, *t* = *t-value*, *Mean*_*Control*_ and *Mean*_*Treatment*_ are respectively the means of control (0 h) and shade treatment (1 h or 6 h), *S*_*p*_ is the pooled standard deviation, and *n* is number of samples in control (0 h) or shade treatment (1 h or 6 h) groups. In this case, the number of samples was same in both control (0 h) and shade treatment (1 h or 6 h) groups (*n* = *n*_*Control*_ = *n*_*Treatment*_).

## Supplementary Information

**Additional file 1: **Supplementary Figures (Fig. S1 – S7) and Tables (Table S1 – S5). **Fig. S1.** Hypocotyl cell size of Brassicaceae vegetables under shade. **Fig. S2.** Root growth of Brassicaceae vegetables under shade. **Fig. S3.** Qualification of RNA-seq. **Fig. S4.** Volcano plots of DEGs. **Fig. S5.** 3000 DEGs from three Brassicaceae vegetables. **Fig. S6.** Up-regulated genes from three Brassicaceae vegetables. **Fig. S7.** Down-regulated genes from three Brassicaceae vegetables. **Table S1.** Summary of RNA-seq reads. **Table S2.** De novo transcriptome assembly statistics. **Table S3.** Statistics of unigenes. **Table S4.** Annotation result of various databases. **Table S5.** List of primers used in this study.

**Additional file 2.** Supplementary Dataset 1 indicating GO analysis of 8 DEGs clusters (associated with Fig. S5).

**Additional file 3.** Supplementary Dataset 2 listing 139 and 297 common DEGs (associated with Fig. 3).

**Additional file 4.** Supplementary Dataset 3 listing 95 and 430 common DEGs (associated with Fig. 4).

## Data Availability

All relevant data supporting the conclusions of this article are included in the article and its supplementary files. The raw RNA-seq data obtained in this study were deposited in Sequence Read Archive, NCBI (PRJNA736537).

## Abbreviations

ABA: Abscisic acid
AUX/IAA: Auxin/indole-3-acetic acid protein
B: Blue
BLAST: Basic Local Alignment Search Tool
BR: Brassinosteroids
Chl a/b: Chlorophyll a/b
CS: Choy Sum
DEGs: Differentially expressed genes
EggNOG: Evolutionary genealogy of genes, Non-supervised Orthologous Groups
FR: Far-red
GA: Gibberellin
GO: Gene ontology
HFR1: LONG HYPOCOTYL IN FAR-RED 1
HY5: ELONGATED HYPOCOTYL 5
IAA: Indole-3-acetic acid
IAAs: INDOLE-3-ACETIC ACID INDUCIBLE proteins
JA: Jasmonic acid
KDR: KIDARI
KEGG: Kyoto Encyclopedia of Genes and Genomes
KL: Kai Lan
NCBI: National Center for Biotechnology Information
PAR: Photosynthetic active radiation
PAR: PHY RAPIDLY REGULATED
PC: Pak Choy
PCA: Principal component analysis
Pfam: Protein families
phyA: phytochrome A
phyB: phytochrome B
PIF: PHYTOCHROME-INTERACTING FACTOR
qRT-PCR: quantitative reverse transcription polymerase chain reaction
R: Red
RNA-seq: RNA sequencing
SA: Salicylic acid
SAR: Shade avoidance response
SAS: Shade-avoidance syndrome
SAUR: SMALL AUXIN UP-REGULATED RNA
TAA: TRYPTOPHAN AMINOTRANSFERASE OF ARABIDOPSIS1
TIR1: TRANSPORT INHIBITOR RESISTANT 1
TMM: Trimmed Mean of M-values
YUC: YUCCA
