# Supplementary material for: Comparative phenotypic and transcriptomic analyses unravel conserved and distinct mechanisms underlying shade avoidance syndrome in Brassicaceae vegetables
Source: BMC Genomics. 2021 Oct 25;22:760. doi: 10.1186/s12864-021-08076-1 (PMC8546956; doi:10.1186/s12864-021-08076-1)
Supplement: Supplementary file 1 — Additional file 1: Supplementary Figures (Fig. S1 – S7) and Tables (Table S1 – S5). Fig. S1. Hypocotyl cell size of Brassicaceae vegetables under shade. Fig. S2. Root growth of Brassicaceae vegetables under shade. Fig. S3. Qualification of RNA-seq. Fig. S4. Volcano plots of DEGs. Fig. S5. 3000 DEGs from three Brassicaceae vegetables. Fig. S6. Up-regulated genes from three Brassicaceae vegetables. Fig. S7. Down-regulated genes from three Brassicaceae vegetables. Table S1. Summary of RNA-seq reads. Table S2. De novo transcriptome assembly statistics. Table S3. Statistics of unigenes. Table S4. Annotation result of various databases. Table S5. List of primers used in this study. [file 12864_2021_8076_MOESM1_ESM.pdf]

Comparative phenotypic and transcriptomic analyses unravel conserved and distinct mechanisms underlying shade avoidance syndrome in Brassicaceae vegetables

Nguyen Hoai Nguyen[1], Benny Jian Rong Sng[1,2], Hock Chuan Yeo[1], and In-Cheol Jang[1,2,*]

[1]Temasek Life Sciences Laboratory, 1 Research Link, National University of Singapore, Singapore 117604, Singapore

[2]Department of Biological Sciences, National University of Singapore, Singapore 117543, Singapore

[*]Correspondence: In-Cheol Jang
Address: Temasek Life Sciences Laboratory, 1 Research Link, National University of Singapore, Singapore 117604
E-mail: jangi@tll.org.sg
Telephone: +65-6872-7409 ; Fax: +65-6872-7007

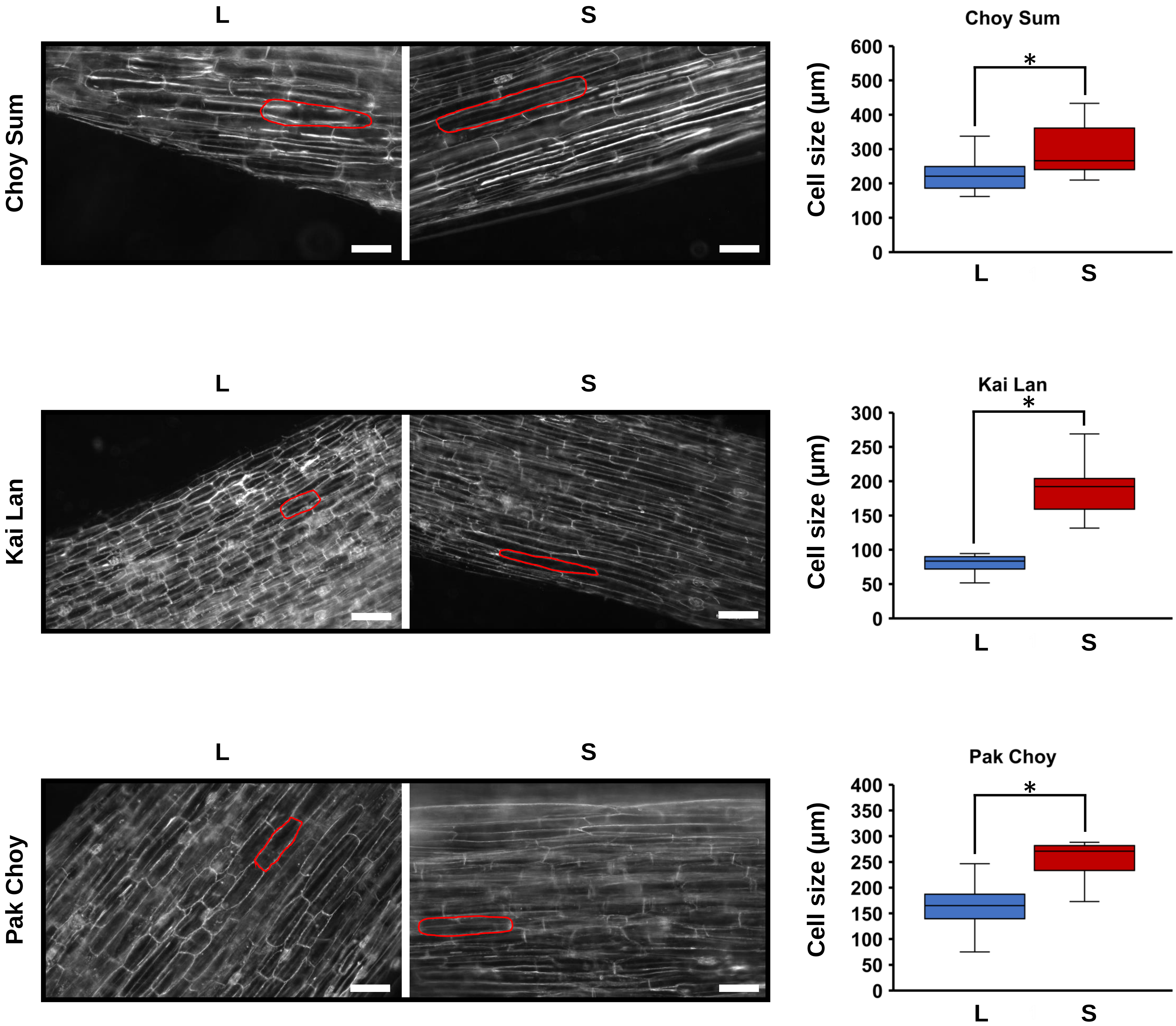

Figure S1. Hypocotyl cell size of Brassicaceae vegetables under shade. Four-day-old Brassicaceae vegetable seedlings grown under normal white light (L) were either kept under L or stimulated to shade (S) for one week. The hypocotyl samples were observed under a light microscope to measure the hypocotyl cell size. Scale = 100 μm. Bars represent average ± SD. n=20. Columns marked with an asterisk differ significantly (*p-value* < 0.05).

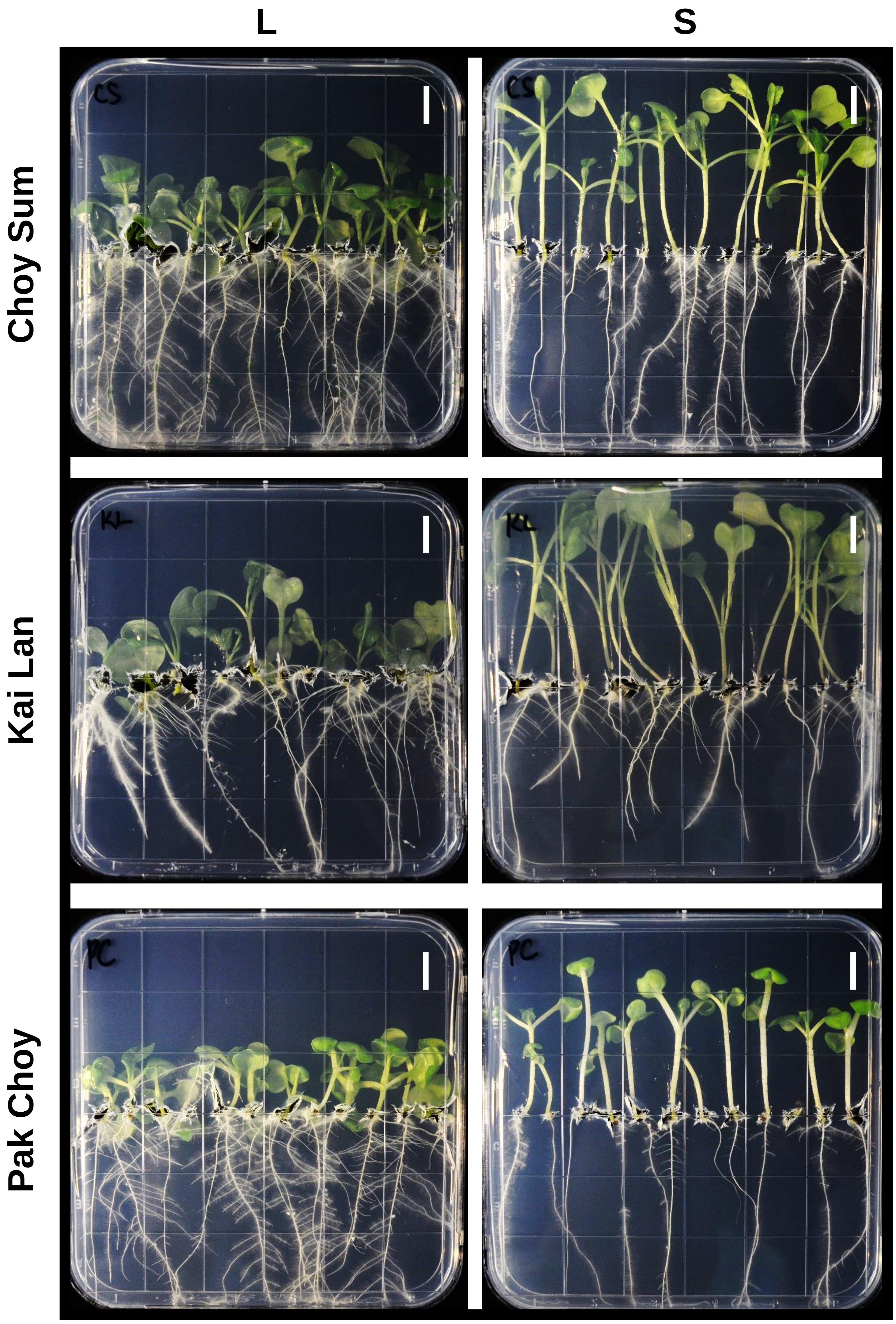

Figure S2. Root growth of Brassicaceae vegetables under shade. Four-day-old Brassicaceae vegetable seedlings grown under normal white light (L) were either kept under L or stimulated to shade (S) for one week. Scale = 1 cm.

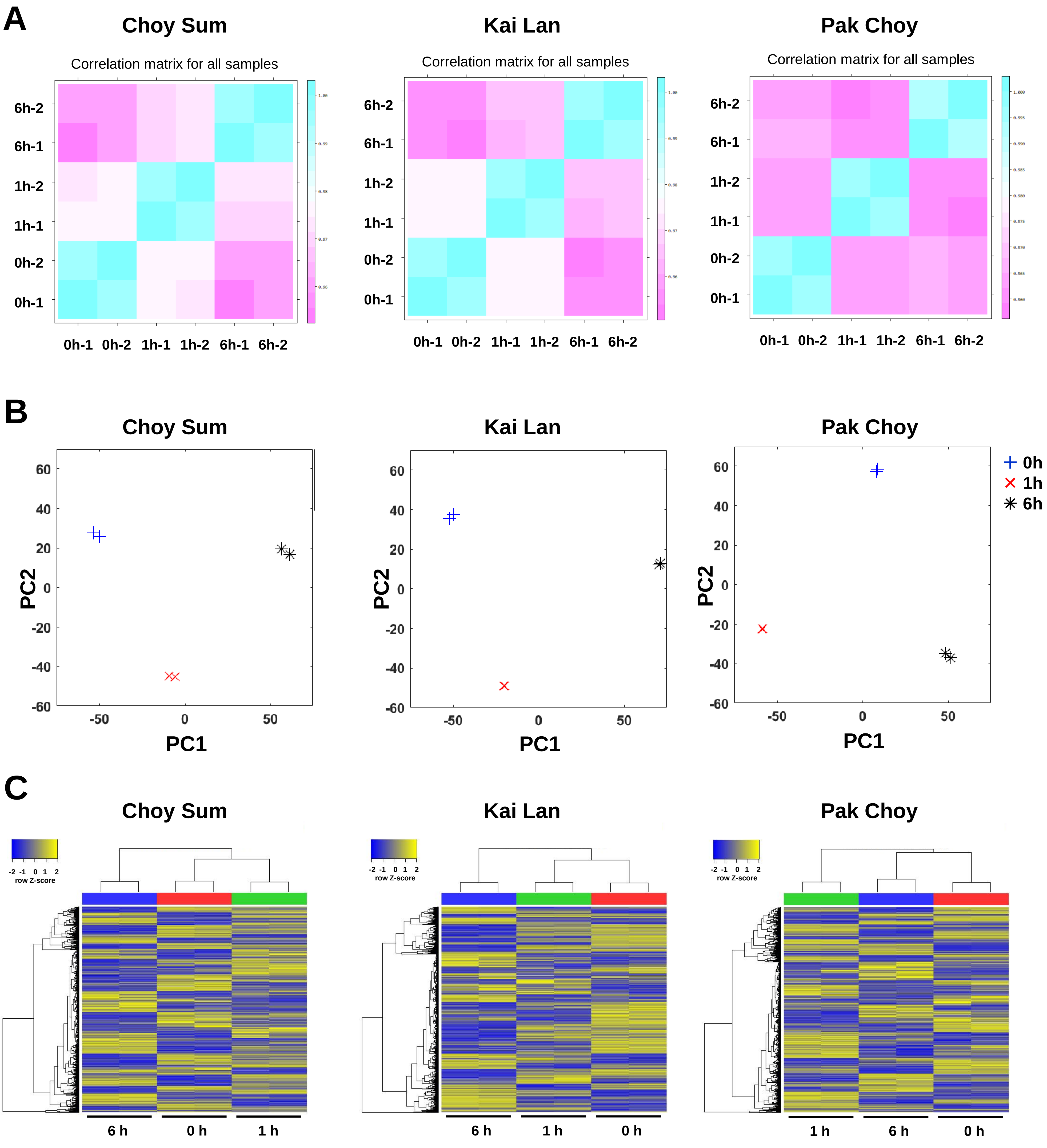

Figure S3. Qualification of RNA-seq. **(A)** Correlation matrix for all samples from each Brassicaceae vegetable. The similarity between samples were obtained through Pearson's coefficient of the normalized value. **(B)** Principal component analysis of RNA-seq. **(C)** Heat-maps showing hierarchical clustering analyses from each vegetable (Choy Sum, Kai Lan, and Pak Choy) treated with shade for 1 and 6 h. Color corresponds to row Z-score.

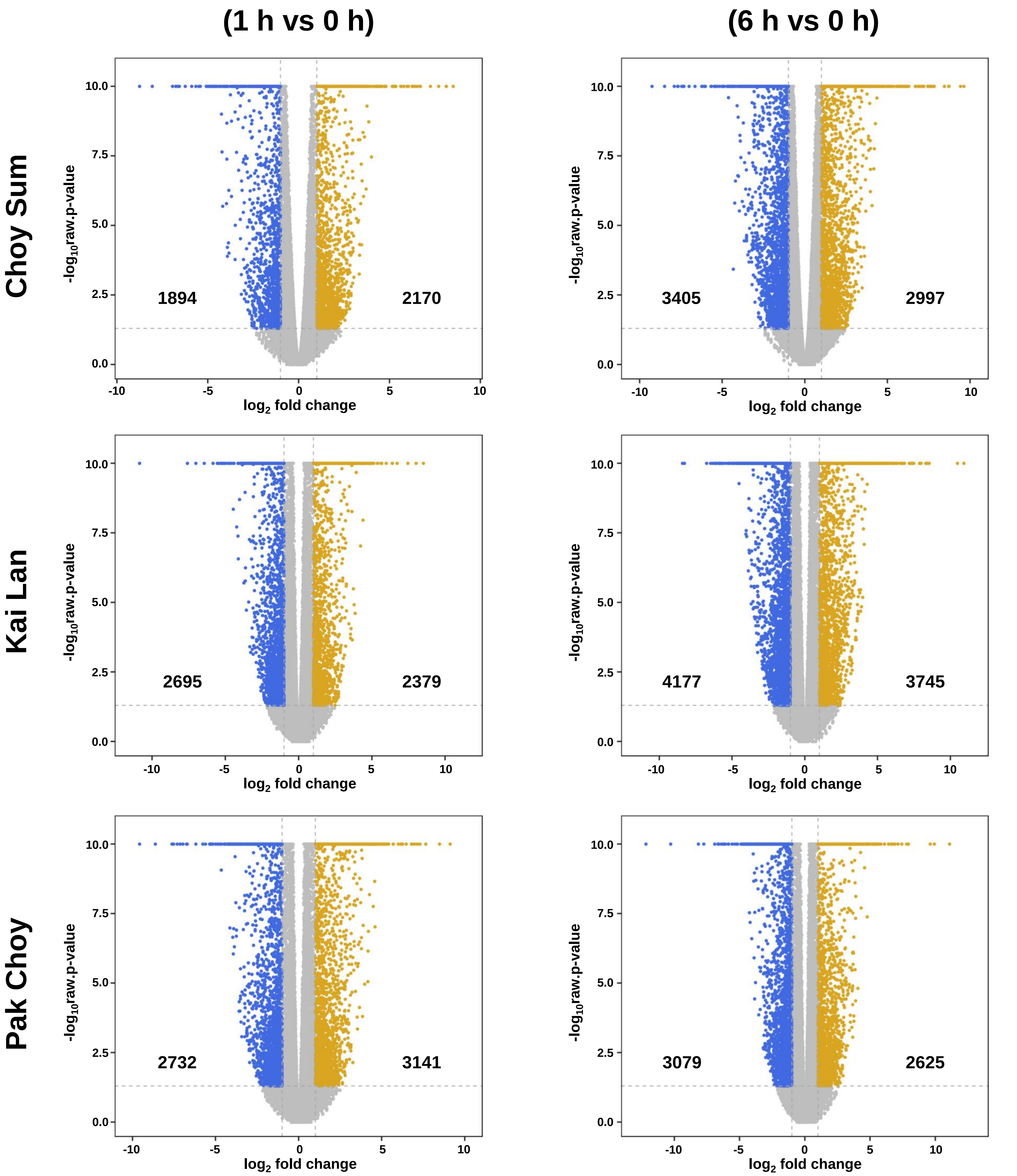

Figure S4. Volcano plots of DEGs. Log2 fold change and *p-value* obtained from the comparison between two groups [(1 h vs 0 h) or (6 h vs 0 h)] were plotted.

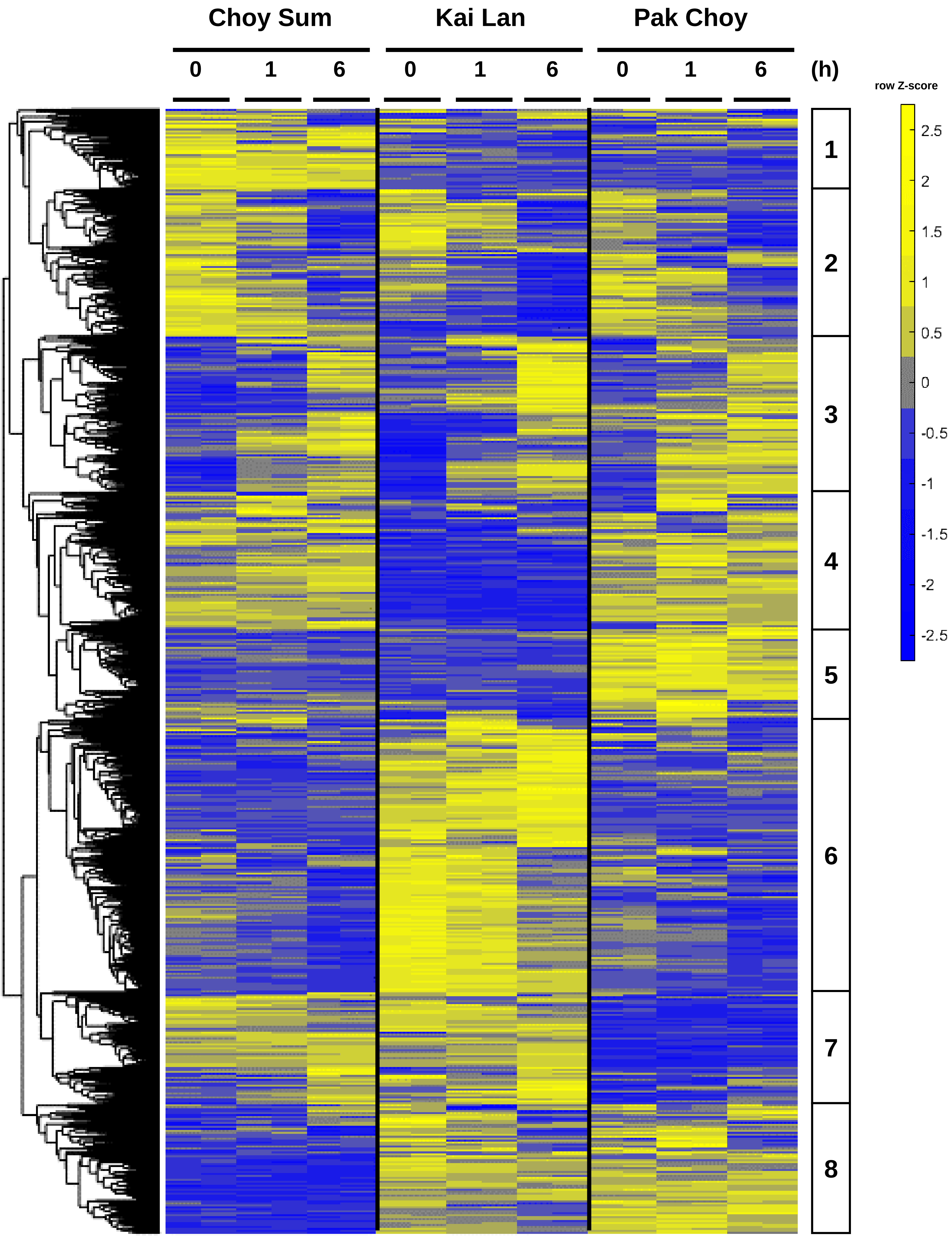

Figure S5. 3000 DEGs from three Brassicaceae vegetables. Heat-maps showed results of hierarchical clustering analyses. Biological replicates in each sample were included in the heat map. Color corresponds to row Z-score.

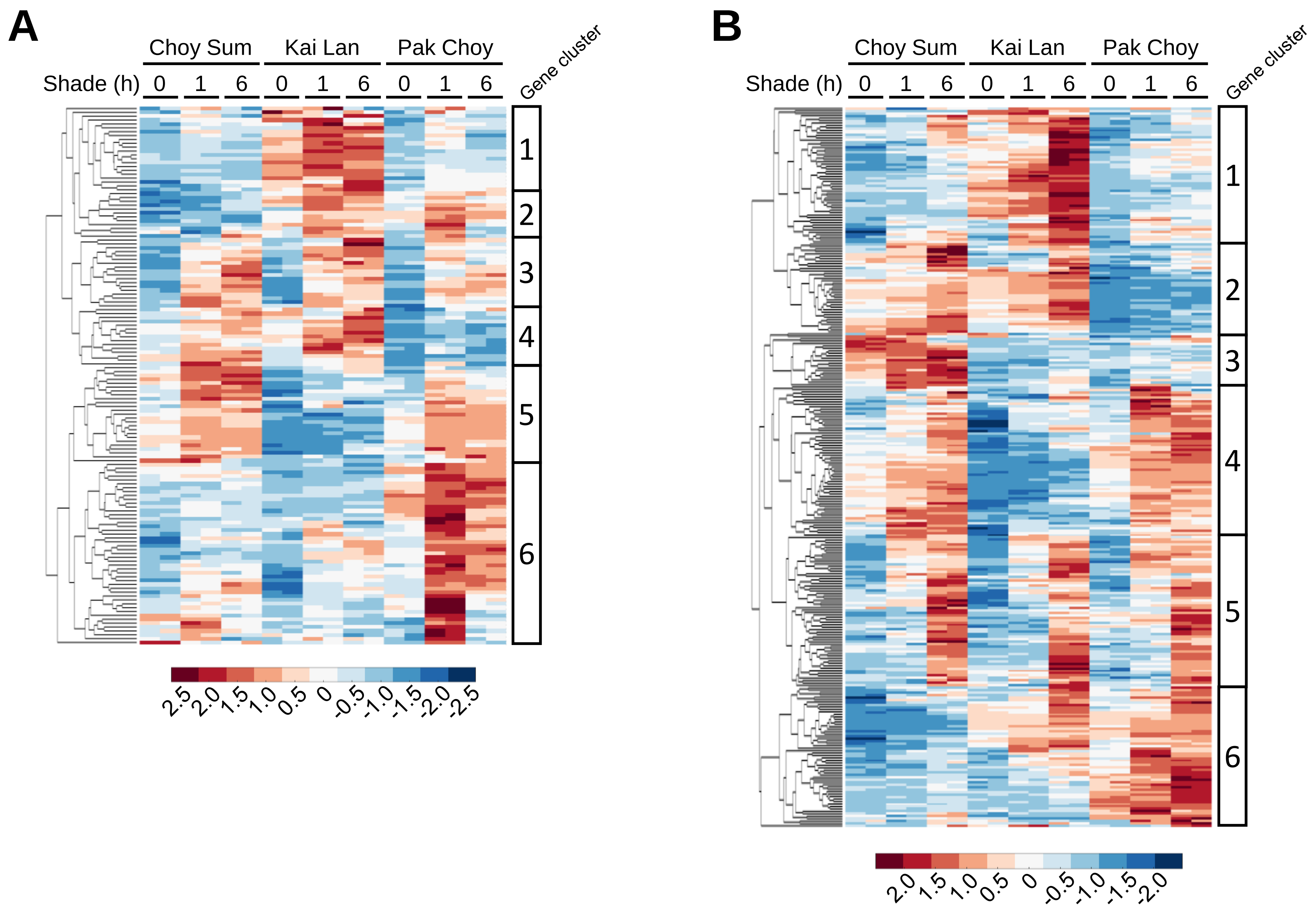

Figure S6. Up-regulated genes from three Brassicaceae vegetables. Expression levels of 139 **(A)** and 297 **(B)** up-regulated genes obtained from merging RNA-seq data (1 h or 6 h vs 0 h) from three Brassicaceae vegetables were shown in heat-map. Both biological replicates in each sample are included in the heat map. Color corresponds to row Z-score.

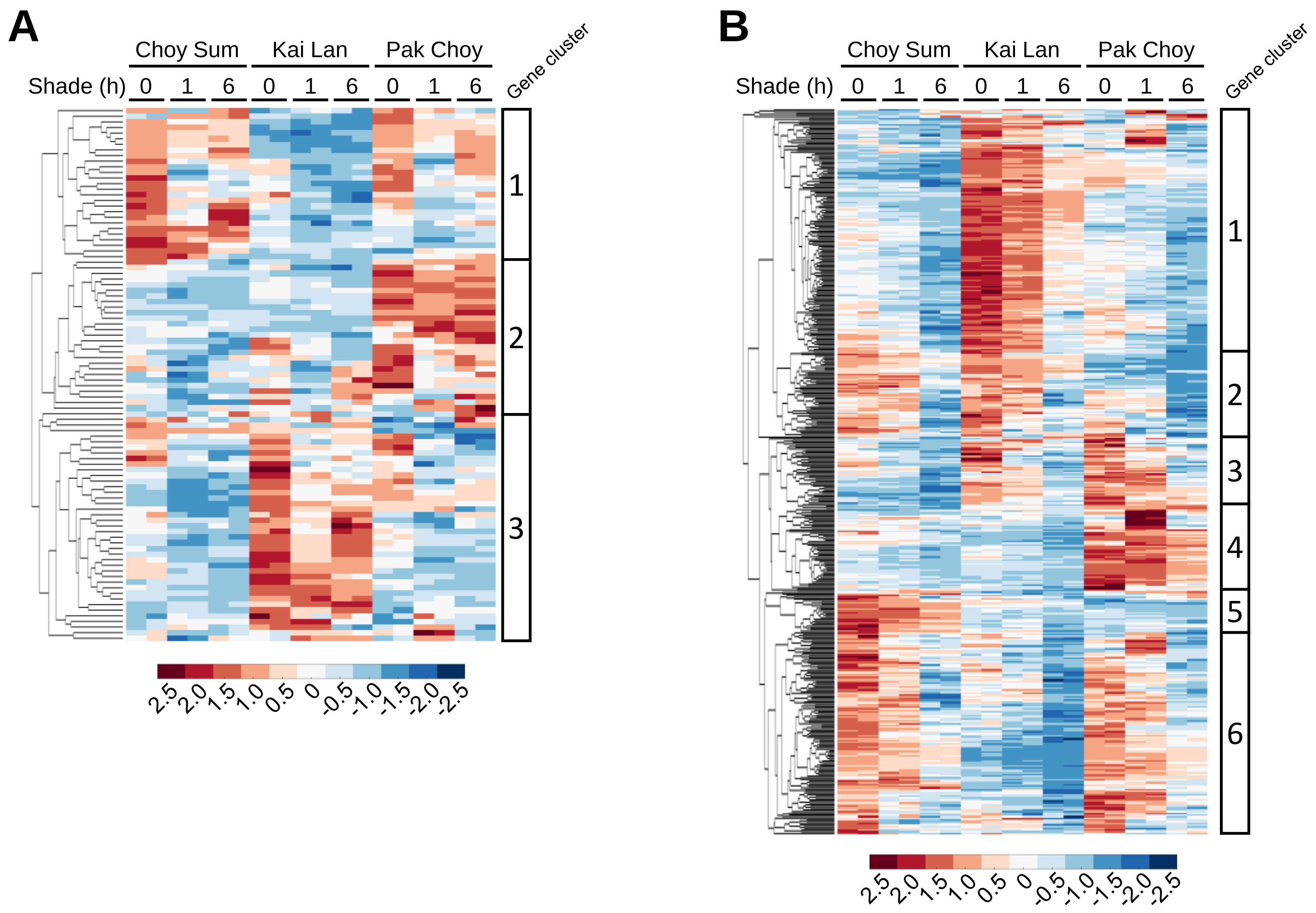

Figure S7. Down-regulated genes from three Brassicaceae vegetables. Expression levels of 95 **(A)** and 430 **(B)** down-regulated genes obtained from merging RNA-seq data (1 h or 6 h vs 0 h) from three Brassicaceae vegetables were shown in heat-map. Both biological replicates in each sample are included in the heat map. Color corresponds to row Z-score.

Table S1. Summary of RNA-seq reads

Sample ID	Total read bases	Total reads	GC(%)	Q20(%)	Q30(%)
**Choy Sum (*Brassica rapa* var. *parachinensis*)**					
**Sample ID**	**Total read bases**	**Total reads**	**GC(%)**	**Q20(%)**	**Q30(%)**
CS-0h-1	17,250,194,343	118,218,864	48.46	98.80	95.64
CS-0h-2	16,820,844,914	115,108,992	48.53	98.75	95.48
CS-1h-1	15,693,178,482	107,272,926	48.13	98.82	95.68
CS-1h-2	16,723,952,806	114,574,768	48.05	98.89	95.89
CS-6h-1	18,943,658,473	129,516,482	47.91	98.84	95.71
CS-6h-1	19,811,644,983	136,299,644	48.06	98.86	95.81
**Kai Lan (*Brassica oleracea* var. *alboglabra*)**					
**Sample ID**	**Total read bases**	**Total reads**	**GC(%)**	**Q20(%)**	**Q30(%)**
KL-0h-1	17,654,244,497	121,034,574	47.77	98.76	95.49
KL-0h-2	16,484,546,091	112,770,724	47.83	98.83	95.71
KL-1h-1	17,123,774,634	117,493,924	48.04	98.77	95.56
KL-1h-2	14,126,964,508	96,865,402	48.08	98.85	95.78
KL-6h-1	16,000,772,585	110,021,082	47.95	98.88	95.86
KL-6h-1	14,935,979,514	101,985,896	48.05	98.81	95.67
**Pak Choy (*Brassica rapa* subsp. *Chinensis*)**					
**Sample ID**	**Total read bases**	**Total reads**	**GC(%)**	**Q20(%)**	**Q30(%)**
PC-0h-1	26,634,583,569	183,728,366	48.09	98.84	95.73
PC-0h-2	16,699,320,699	114,434,606	48.14	98.82	95.69
PC-1h-1	19,035,094,127	130,185,282	48.11	98.81	95.65
PC-1h-2	16,545,871,563	112,917,526	47.98	98.83	95.70
PC-6h-1	19,949,691,348	137,083,706	48.23	98.84	95.73
PC-6h-1	15,693,762,243	107,918,324	48.15	99.30	97.02

Table S2. *De novo* transcriptome assembly statistics

	Choy Sum	Kai Lan	Pak Choy
**Total number of trinity 'genes'**	111,503	127,172	123,223
**Total number of trinity transcripts**	156,234	189,879	175,656
**GC(%)**	42.92	42.60	42.75
**N90 (bp)**	309	317	301
**N80 (bp)**	467	480	454
**N70 (bp)**	689	698	666
**N60 (bp)**	947	944	920
**N50 (bp)**	1,227	1,213	1,196
**N40 (bp)**	1,529	1,504	1,491
**N30 (bp)**	1,877	1,845	1,828
**N20 (bp)**	2,366	2,317	2,307
**N10 (bp)**	3,258	3,173	3,196
**Maximum contig length (bp)**	16,487	16,506	16,457
**Minimum contig length (bp)**	201	201	201
**Median contig length (bp)**	452.0	471.0	441.0
**Average contig length (bp)**	768.97	776.57	751.09
**Total assembled bases**	120,138,595	147,453,755	131,934,297

Table S3. Statistics of unigenes

	Choy Sum	Kai Lan	Pak Choy
**Total number of 'unigenes'**	86,733	99,896	95,984
**GC(%)**	42.39	41.96	42.11
**N90 (bp)**	282	285	271
**N80 (bp)**	400	285	378
**N70 (bp)**	584	401	547
**N60 (bp)**	856	576	803
**N50 (bp)**	1,170	827	1,121
**N40 (bp)**	1,509	1,463	1,463
**N30 (bp)**	1,873	1,828	1,832
**N20 (bp)**	2,372	2,317	2,333
**N10 (bp)**	3,295	3,186	3,242
**Maximum contig length (bp)**	16,487	16,506	16,457
**Minimum contig length (bp)**	201	201	201
**Median contig length (bp)**	391.0	399.0	374.0
**Average contig length (bp)**	707.93	702.54	679.97
**Total assembled bases**	61,400,836	70,180,634	65,265,787

Table S4. Annotation result of various databases

	Choy Sum	Kai Lan	Pak Choy
**Total ‘unigenes'**	86,733	99,896	95,984
**GO**	49,400 (56.96%)	53,298 (53.35%)	51,811 (53.98%)
**UniProt**	40,257 (46.41%)	42,794 (42.84%)	42,373 (44.15%)
**NR**	56,257 (64.86%)	62,539 (62.6%)	59,871 (62.38%)
**Pfam**	37,830 (43.62%)	41,060 (41.1%)	39,574 (41.23%)
**EggNOG**	54,746 (63.12%)	59,478 (59.54%)	57,899 (60.32%)
**NT**	72,007 (83.02%)	80,857 (80.94%)	80,482 (83.85%)
**KEGG**	54,370 (62.69%)	59,507 (59.57%)	57,454 (59.86%)

Table S5. List of primers used in this study

**For RT-qPCR**			
**Name**	**Forward primer (5′-3′)**	**Reverse primer (5′-3′)**	**Plant species**
BraACTIN2	CATGTTCACCACAACAGCCG	AGTCTCCATCTCCTGCTCGT	Choy Sum, Kai Lan, and Pak Choy
BraYUC8	TGAGAGAGTGATGCCGGAGA	TGTAATCACAGGCGTGGACC	Choy Sum, Kai Lan, and Pak Choy
BraYUC9	GGAGTCCCATTCGTTGTGGT	AGCCTGTCGTACGTTCGTTT	Choy Sum, Kai Lan, and Pak Choy
BraIAA29	GGACTTAGACCGTCATCGTCA	GGTAATAGCCAGTCGCCCTC	Choy Sum, Kai Lan, and Pak Choy
BrPAR1	TTTGAGCGCAGAACCAAACG	AATCCTCTGCAACGCCTCAA	Choy Sum and Pak Choy
BoPAR1	GGTGGTTTCGAACGCAGAAC	TTCTTCTTGGCCGGAGACAC	Kai Lan
BrPAR2	ACGTCCCACTGGTTTCAAGC	GCTCTCTCTTACACTCGCCG	Choy Sum
BoPAR2	TCAAGCGCAGAACCAGACAA	GCCGCTCCTCCTGGTACTAT	Kai Lan
BraGH3.3	CCATCACCGAGTTCCTGACC	AAAAGCTGACGTCGGTCCAT	Choy Sum, Kai Lan, and Pak Choy
BraIAA19	AACGGAGCTGAGACTTGGAC	GCCGCTCTCACATTGTTCAC	Choy Sum, Kai Lan, and Pak Choy
BraSAUR21	GGTGTACGTAGGAGAGAG	TGGAGCCTAGACGTTA	Choy Sum, Kai Lan, and Pak Choy
